# Influence of Extraction Solvent on Nontargeted Metabolomics Analysis of Enrichment Reactor Cultures Performing Enhanced Biological Phosphorus Removal (EBPR)

**DOI:** 10.3390/metabo11050269

**Published:** 2021-04-26

**Authors:** Nay Min Min Thaw Saw, Pipob Suwanchaikasem, Rogelio Zuniga-Montanez, Guanglei Qiu, Ezequiel M. Marzinelli, Stefan Wuertz, Rohan B. H. Williams

**Affiliations:** 1 Singapore Centre for Environmental Life Sciences Engineering, Nanyang Technological University, Singapore 637551, Singapore; nsaw@ntu.edu.sg (N.M.M.T.S.); roge.zun@gmail.com (R.Z.-M.); qiugl@scut.edu.cn (G.Q.); e.marzinelli@sydney.edu.au (E.M.M.); swuertz@ntu.edu.sg (S.W.); 2 Singapore Phenome Centre, Nanyang Technological University, Singapore 636921, Singapore; tun_be@hotmail.com; 3 Department of Civil and Environmental Engineering, One Shields Avenue, University of California, Davis, CA 95616, USA; 4 School of Life and Environmental Sciences, The University of Sydney, Sydney, NSW 2006, Australia; 5 School of Civil and Environmental Engineering, Nanyang Technological University, Singapore 639798, Singapore; 6 Singapore Centre for Environmental Life Sciences Engineering, National University of Singapore, Singapore 117456, Singapore

**Keywords:** microbial ecology, activated sludge, untargeted metabolomics, extraction, polyphosphate-accumulating organisms, enhanced biological phosphorus removal, ultra-high performance liquid chromatography, mass spectrometry

## Abstract

Metabolome profiling is becoming more commonly used in the study of complex microbial communities and microbiomes; however, to date, little information is available concerning appropriate extraction procedures. We studied the influence of different extraction solvent mixtures on untargeted metabolomics analysis of two continuous culture enrichment communities performing enhanced biological phosphate removal (EBPR), with each enrichment targeting distinct populations of polyphosphate-accumulating organisms (PAOs). We employed one non-polar solvent and up to four polar solvents for extracting metabolites from biomass. In one of the reactor microbial communities, we surveyed both intracellular and extracellular metabolites using the same set of solvents. All samples were analysed using ultra-performance liquid chromatography mass spectrometry (UPLC-MS). UPLC-MS data obtained from polar and non-polar solvents were analysed separately and evaluated using extent of repeatability, overall extraction capacity and the extent of differential abundance between physiological states. Despite both reactors demonstrating the same bioprocess phenotype, the most appropriate extraction method was biomass specific, with methanol: water (50:50 *v*/*v*) and methanol: chloroform: water (40:40:20 *v*/*v*/*v*) being chosen as the most appropriate for each of the two different bioreactors, respectively. Our approach provides new data on the influence of solvent choice on the untargeted surveys of the metabolome of PAO enriched EBPR communities and suggests that metabolome extraction methods need to be carefully tailored to the specific complex microbial community under study.

## 1. Introduction

Untargeted metabolome profiling is becoming more commonly deployed in the study of complex microbial communities and microbiomes, and this general approach will likely play an increasingly important role in future microbial ecology studies in both host-associated and environmental settings [1].

Performing metabolomics on microbial community samples remains challenging [2], in part due to the fact that multiple member species could produce or utilise a given compound [3] and in part due to the high proportion of unannotated mass features that will be detected [4]. Solving these problems will require substantial efforts in the areas of data analysis and interpretation [5,6], new modelling methods [7,8] and large-scale compound annotation [4,9].

Further technical challenges exist due to potential differential extractability among the varied cell types of member species of a microbial community, as well as in the complex biomass morphology that can be encountered, such as flocs or granules [10], and which requires the use of extensive mechanical disruption when performing biomolecular extractions. This level of complexity suggests that extraction procedures suitable for specific kinds of microbial community biomass may need to be carefully evaluated.

The choice of an efficient and reproducible metabolite extraction and sample preparation method is extremely important in metabolomics studies, as it is known to influence the observed metabolite features and the subsequent biological interpretation of the data [11]. An appropriate extraction method should be simple and fast in order to provide an accurate snapshot of the metabolome and avoid possible enzymatic or chemical degradation during the procedure [12]. Ideally, metabolome analysis should try to extract as many classes as possible of metabolites, with both polar (e.g., methanol or ethanol) and non-polar (e.g., ethyl acetate, hexane and chloroform) solvents being targeted [5,12,13,14].

In this study, we seek to examine these issues in the context of complex microbial community biomass present in laboratory-scale wastewater treatment bioreactors. Complex microbial communities resident in wastewater treatment plants are responsible for the removal of excess nutrients, organic compounds and specific pollutants in waste sources arising from domestic, industrial, health-related and agricultural ecosystems [15]. Despite decades of study, considerable gaps remain between the knowledge of the bioprocess engineering aspects of wastewater treatment and the underlying microbial ecophysiology of activated sludge communities, which may limit stability, optimisation and future innovation in water reclamation technologies [10,15]. Recent use of omics technologies has provided many new insights, but to date, most activity has been focused on using whole community metagenomics and metatranscriptomics, with very limited use of community-wide metabolomics studies [16].

Enhanced biological phosphorus removal (EBPR) is a wastewater treatment bioprocess that permits the removal of excessive phosphorus from soluble wastewater without the use of chemical precipitants [17] and is therefore of considerable interest in regard to the deployment of sustainable solutions in dealing with human waste [18,19,20]. The EBPR bioprocess is associated with cyclic anaerobic–aerobic physiological states [10], during which a class of microbes, referred to as polyphosphate-accumulating organisms, or PAOs, can undertake excessive release and uptake of soluble phosphorus in the wastewater. Release of phosphate from cells is observed in the anaerobic state, while uptake is observed in the aerobic state [10]. Over multiple cycles, biomass increases during the aerobic phase, and synchronised removal of biomass as it settles out of suspension leads to the reduction of phosphorus concentration in the wastewater [10]. 

The EBPR bioprocess can be studied directly in full scale systems [21,22] and has also been extensively studied in laboratory-scale continuous culture enrichment bioreactors, which have permitted the identification of the species that are reasonable for excessive phosphorus uptake (discussed in [17,22,23]), the recovery of their genomes [24,25,26] and exploration of their ecophysiology [27,28], as well as making use of these PAO enrichment to evaluate or bioprocess refinements that can then be tested in full scale EBPR systems [21]. 

Furthermore, such wastewater treatment communities can form an ideal model system for studying more fundamental issues in microbial ecology and ecosystems biology [29]. However, as discussed above, there are little untargeted metabolomics data available in such communities, and in particular, there are no insights available on suitable metabolome extraction procedures for the suspended floccular biofilms that typically constitute the biomass of these wastewater treatment microbial communities [10].

Here, we report the results of an untargeted metabolomics analysis undertaken in two laboratory-scale activated sludge reactors whose microbial communities are enriched for PAO species, with each exhibiting the EBPR phenotype. The specific aim of this study is to explore solvent conditions suitable for sample preparation of microbial cells in activated sludge bioreactor communities that are performing enhanced biological phosphorus removal.

Metabolites from two different EBPR reactor microbial communities were extracted using different solvent mixtures and analysed by ultra-performance liquid chromatography mass spectrometry (UPLC-MS). As for the majority of naturally occurring microbial communities, these samples will contain a high species diversity and a substantive proportion of unculturable members, and accordingly, there is very limited a priori insight into what extraction solvents may be suitable (this contrasts with the situation encountered when working with well-established, culturable microbial model organisms e.g., as reviewed by Pinu et al. [12]). We therefore studied one non-polar solvent and several variations of polar extraction solvents, the latter was chosen to gain insight into the sensitivity of mixture variation. We evaluated how these different solvent mixtures impacted (1) overall variability of LC-MS feature profiles; (2) the repeatability of individual LC-MS feature measurements; and (3) the overall extraction capacity and diversity, assessed at both LC-MS feature and annotated compound level. Additionally, we considered the influence of solvent type on (4) the extent of LC-MS feature differential abundance between physiological states associated with phosphate release and uptakes stages of the EBPR cycle, and (5) the potential impact on biological interpretation, as surveyed by known metabolic pathways that are implicated by the set of annotated LC-MS features. 

## 2. Results

We sampled two laboratory enrichment bioreactor communities operated using two different protocols, each designed to enrich different PAO species, from here on described as Reactor A and Reactor B (see Section 4.1). Although these bioreactors both exhibit the same functional phenotype (EBPR), their distinct physico-chemical feeding histories and species composition implies they are not biological replicates, and, therefore, our analysis strategy is designed for each reactor independently.

**Figure 1:**
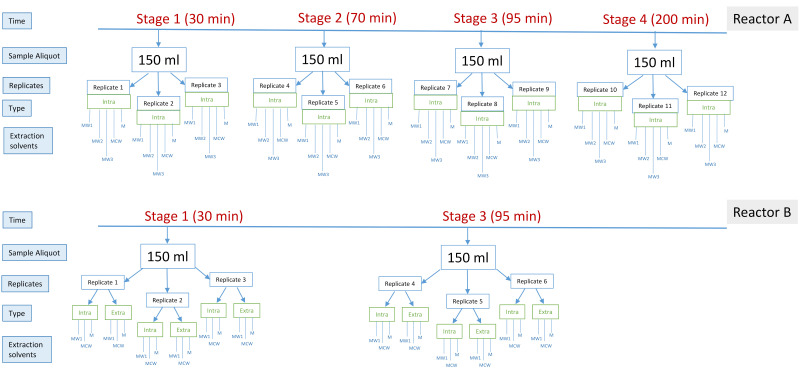
Schematic diagram of experimental design for (upper panel) Reactor A and (lower panel) Reactor B.

We obtained samples from each reactor at multiple time points during a single 6 h cycle study (see Figure 1 and Section 4.2). In Reactor A, we took samples at four timepoints across one EBPR cycle, with two samples taken from the anaerobic phase and two from the aerobic phase (Figure 1, Reactor A), whereas in Reactor B, a single timepoint from each phase was obtained (Figure 1, Reactor B). In the case of Reactor A, intracellular biomass was tested with five different extraction solutions for use with untargeted UPLC-MS, specifically, pure methanol (M), methanol: water (50:50 *v*/*v*) (MW1), methanol: water (60:40 *v*/*v*) (MW2), methanol: water (80:20 *v*/*v*) (MW3) and methanol: chloroform: water (40:40:20 *v*/*v*/*v*) (MCW). For Reactor B, we tested both intracellular biomass and extracellular supernatant using three extraction solutions, namely M, MW1 and MCW (Figure 1B and Section 4.2).

To gain insight into the microbial community composition of each reactor, we examined 16S amplicon sequencing data (see Methods: 16S Amplicon Sequencing Data) that were obtained from each reactor community as part of separate studies and matched as close as possible in time (one day apart in the case of Reactor B, and the following month in the case of Reactor A). In Reactor A, a total of 242 amplicon sequence variants (ASV) were observed, with 50% of community composition (as measured by ASV relative abundance) being accounted for by 11 taxa (Supplementary Data File 1). For Reactor B, from a sample obtained the day after the sampling event used for the present study, a total of 1234 ASV were observed, with 5 taxa accounting for 50% of community composition (Supplementary Data File 1). These sets of most abundant taxa had two taxa in common, namely genus *Marmoricola* and genus *Nocardioides*, highlighting the substantive differences in taxonomic composition between the two microbial communities.

### 2.1. General Profiles of LC-MS Features in Lab-Scale EBPR Bioreactor Microbial Communities

**Figure 2:**
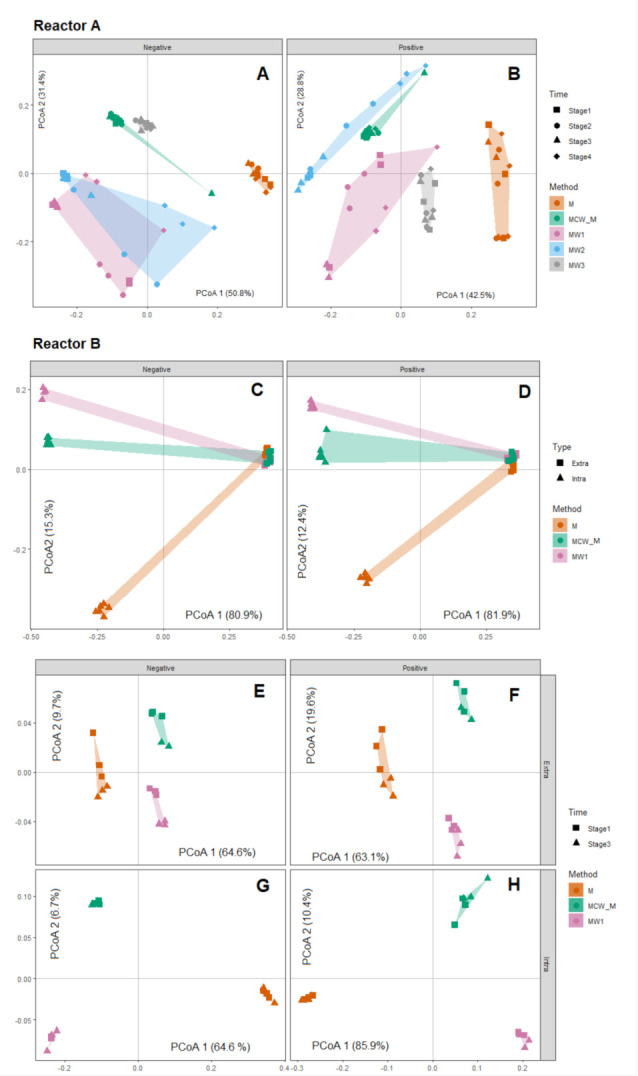
Principal coordinates analysis (PCoA) of untargeted metabolite profiles of Reactor A (panels A,B) and Reactor B (panels C–H) extracted by different extraction solvents. (**A**) PCoA analysis of negative mode data, with samples from each of the five solvent methods delineated by coloured convex hulls and samples from each of the four time points by different symbols. (**B**) Positive mode data from Reactor A, with the same formatting as for panel A; (**C**) PCoA analysis of negative mode data from Reactor B, with samples from each of the three solvent methods delineated by coloured convex hulls and samples from each compartment (intra- or extra-cellular) delineated by symbol; (**D**) PCoA analysis of positive mode data from Reactor B, with the same formatting; (**E**–**H**) within—compartment PCoA analysis for extracellular negative (**E**) and positive (**F**) mode data, and intracellular negative (**G**) and positive (**H**) data; samples from each of the three solvent methods delineated by coloured convex hulls and samples from each experimental stage (time) delineated by symbols.

We first examined the overall differences in LC-MS feature profiles among samples from each reactor using principal coordinate analysis (PCoA), with samples categorised by different extraction solvents and, additionally, in the case of Reactor B, by compartment (intracellular or extracellular) (Figure 2). The PCoA-based visualisation was augmented by use of the statistical inference procedure PERMANOVA [30,31] (Table 1). These multivariate analyses were applied separately to data obtained from polar (M, MW1-3 and MCW_M) and non-polar (MCW_C) solvents. In Reactor A, we observed that different extraction solvents demonstrated distinct LC-MS feature profiles in both positive (Figure 2A and Table 1; *p* = 0.0001 PERMANOVA) and negative ionisation mode (Figure 2B and Table 1; *p* = 0.0001 PERMANOVA), and that samples from the same extraction solvents were generally separated in the plane of the first and second PCoA scores. In both positive and negative modes, samples from the M method were most distinct from the other four methods, being completely distinct on the first PCoA axes. In positive mode, samples from MCW and MW2 methods tended to be more closely related, whereas negative mode MW1 and MW2 showed both a similarity to MCW (Figure 2A). A statistically significant interaction between solvent type and time were observed from both acquisition modes (*p* = 0.003; positive mode; *p* = 0.0001; negative mode, Table 1). Post hoc analysis of pairwise combinations of solvent type generally differed from each other at all time points (Supplementary Data File 2), consistent with the main effect result for the solvent method. In negative mode, samples from MW1 and MW2 were relatively similar to one another, particularly at stages 1, 2 and 4, with the other three methods being distinct. In positive and negative mode, differences among time points were generally driven by differences between the last time point (stage 4) and other time points, and this was consistent for most solvent types (see post hoc analysis summarised in Supplementary Data Files 2–3). Collectively, these observations suggest that solvent type exerts a strong influence on observed LC-MS feature profiles, but also that there is a high degree of sensitivity in detecting differences in physiological state, as induced over time in the case of the present study.

For Reactor B, the LC-MS feature profiles of extracellular samples were completely distinguishable from those of intracellular samples (Figure 2C,D). In the extracellular case, samples were tightly clustered in both positive (Figure 2C) and negative mode (Figure 2D) and showed a much smaller range of variation compared to intracellular LC-MS feature profiles. Compared within compartment type, solvent method M showed a greater degree of difference to both MCW and MW1, in the case of both extracellular (Figure 2E,F) and intracellular (Figure 2G,H) extractions. Despite significant interactions between solvent type and compartment, and compartment and time (Table 1), all solvent types differed from each other for both intracellular and extracellular samples. Similarly, extracellular and intracellular samples differed from each other, and this difference was consistent for all solvent types and all time points (Supplementary Data Files 4–5). This was consistent for positive and negative modes (Table 1).

The data from the non-polar phase derived from the MCW extraction method were also investigated for sample-to-sample variation of each time point in Reactor A and each sample type in Reactor B. From the PCA sample score plot (Figure S1A,C), the MCW does not give a clear separation among the different time points in Reactor A. However, MCW extraction for Reactor B apparently showed a large variation between extra- and intra-cellular non-polar molecules with overlapping data points between two different stages of time during the EBPR cycle (Figure S1B,D).

### 2.2. Extraction Capacity

We next examined the influence of solvent mixture on the overall metabolite extraction capacity by examining (1) the total number of LC-MS features and the number of annotated compounds (defined against KEGG compound identifiers; see Section 4) (summarised for Reactor A and Reactor B in Table 2) and (2) distribution of LC-MS features across both retention–time and mass–charge ratio axes (Figures S2–S5), under the assumption that a more uniform distribution of LC-MS features across one or both of these axes will suggest the solvent that is capable of obtaining a wide variety of compounds from a given sample type.

In Reactor A, MW1 generated the largest number of LC-MS features, in both ionisation modes (Table 2), closely followed by MW2, MW3 and the aqueous fraction of MCW. The remaining two methods showed a drop in the number of LC-MS features, with M generating around 80–85% of the total number of LC_MS features captured by each of the preceding four methods, while the chloroform fraction generated 25–27%. For positive mode data, MW1 generated the most uniform distribution of LC_MS features across the retention–time axis, followed by MW2 and then MCW, whereas MW3 and M gave the lowest diversity of LC_MS features (Figure S4). In negative mode, the variation in diversity on both axes showed less marked differences, most notably in the case of the m/z axis (Figure S5). For the solvents tested on the Reactor A biomass, the proportion of putatively annotated compounds detected was on average 38.0% (range: 24.7–43.6%) of the total number of LC-MS features identified from the positive mode data, with a slightly higher proportion (41.1%, range: 12.9–51.0%) being observed from negative mode data. In both modes, the smallest proportion was observed in data obtained from the chloroform fraction of the MCW solvent mixture (MCW_C; Table 2). 

For Reactor B, the M, MW1 and the aqueous fraction of MCW generated similar numbers of LC-MS features in positive mode (Table 2) but generated higher numbers in negative mode, with MW1 and the MCW aqueous fraction generating the largest numbers of LC-MS features followed by M. Interestingly, the chloroform fraction of the MCW extraction generated around double the number of LC-MS features in positive mode compared to any other solvent type (Table 2). In positive mode, and to a lesser degree in negative mode, variation in the uniformity of LC-MS feature distribution over retention time and m/z was less evident than in the case of Reactor A. On average, 64.8% (range: 28.9–79.3) and 52.5% (range: 22.8–65.0) of LC-MS features held putative identifications to compounds, in positive mode and negative mode datasets, respectively.

For data obtained from the chloroform fraction of the MCW solvent mixture (MCW_C), the number of annotated compounds was low (4% in positive mode, and 11% in negative mode), whereas from the three other solvent mixtures, the number of annotated compounds was on average around 26% of the number of LC-MS features in both modes (26.2%, range: 24.9–28.7% for positive mode, and 26.7%, range: 25.1–28.2% in the case of negative mode). The higher proportion of LC-MS features and putative annotations from the non-polar solvent is consistent with a higher lipid fraction in the biomass from Reactor B than from that of Reactor A.

For reference, the total ion chromatograms (TIC) of LC-MS profiles from each extraction method and ionisation mode in both reactors are also provided in Figures S6 and S7.

### 2.3. Evaluation of Method Repeatability 

**Figure 3:**
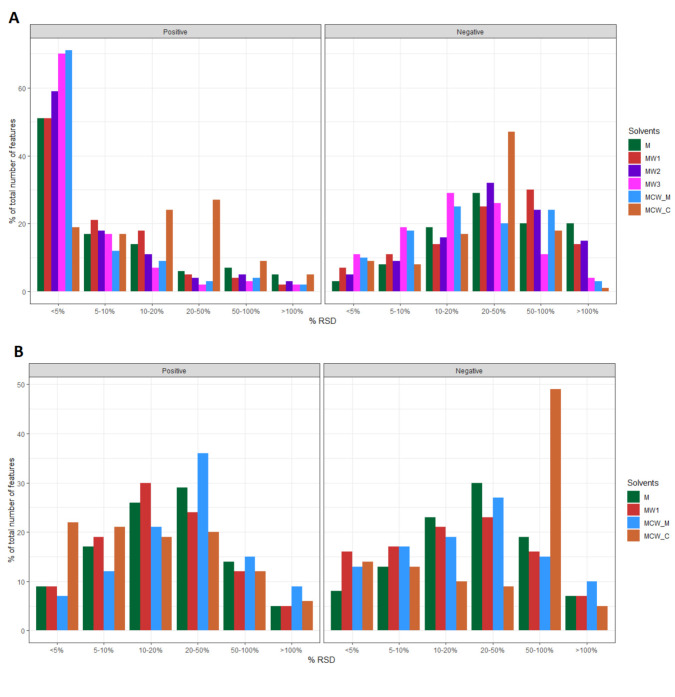
Distribution of relative standard deviation (RSD%) of metabolite features (*m*/*z*) among different solvent extractions for each reactor and ionisation mode. (**A**) Reactor A in both positive (left panel) and negative (right panel) ionisation modes; (**B**) Reactor B in positive (left panel) and negative (right panel) ionisation modes.

To examine whether the use of different solvents influenced the measurement repeatability of individual LC-MS features, the distribution of relative standard deviation (RSD) of detected abundance of all LC-MS features was examined (Figure 3). Compared to all other extraction solvents used in Reactor A, the aqueous fraction of the MCW extraction solvent resulted in less analytical variation among other solvents, with more than 60% of total LC-MS features detected in positive ionisation mode showing an RSD value below 5%. Data from the chloroform fraction of MCW showed the highest degree of variability (Figure 3A), with the remaining three methods showing variability closer to MCW_M. In negative mode, the overall degree of variability was higher, with MW3 giving the lowest overall variability. Similar results were observed for Reactor B, with the majority of LC_MS features showing RSD values in the 10–50% range. In contrast to the case of Reactor A, the MCW_M and MCW_C methods demonstrated the lowest overall variability for positive mode data suggesting that a methanol: chloroform: water solvent mixture would be the ideal solvent for the extraction of metabolites in Reactor B (Figure 3B).

### 2.4. Biological Level Interpretation and Differentiation of Physiological States

In the final part of our analysis, we explore how the use of different solvent types can potentially impact two aspects of biological-level interpretation that are commonly encountered in the literature, specifically by examining (1) which metabolic pathways are implicated by the set of putative compound identification and (2) the number of LC-MS features that are differentially abundant between the different physiological states (anaerobic and aerobic, respectively) that the microbial community experiences over time, and that are coupled to the excessive phosphorus release and uptake phenomena that are characteristic of the EBPR bioprocess. 

**Figure 4:**
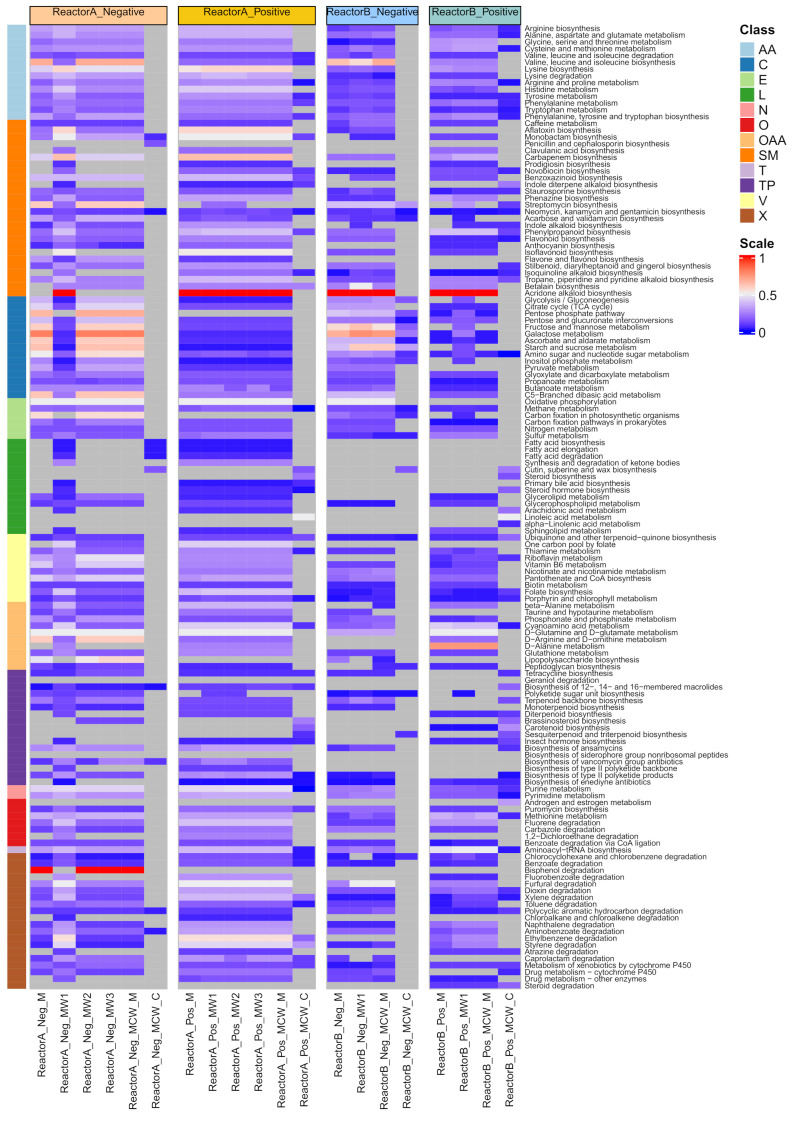
Heatmap representation showing recovery of canonical metabolic pathways (KEGG) as identified from putative compound identifications (KEGG compound identifier), categorised by reactor-of-origin, type of solvent mixture and acquisition mode. The colour scale expresses the proportion of member compounds identified (number of detected member compounds divided by the total number of member compounds, as defined by KEGG). Pathways for which no member compounds were detected are shown in grey. Pathways are organised by their KEGG class classification and abbreviated as follows: *AA*: amino acid metabolism; *C*: carbohydrate metabolism; *E*: energy metabolism; *L*: lipid metabolism; *N*: nucleotide metabolism; *O*: others; *OAA*: metabolism of other amino acids; *SM*: biosynthesis of other secondary metabolites; *T*: translation; *TP*: metabolism of terpenoids and polyketides; *V*: metabolism of cofactors and vitamins; *X*: xenobiotics biodegradation and metabolism. Solvent mixtures denoted as: *M*: pure methanol; *MW1*: methanol: water (50:50 *v*/*v*); *MW2*: methanol: water (60:40 *v*/*v*); *MW3*: methanol: water (80:20 *v*/*v*); *MCW_M*: methanol: chloroform: water (40:40:20 *v*/*v*/*v*)_aqueous fraction; *MCW_C*: methanol: chloroform: water (40:40:20 *v*/*v*/*v*)_chloroform fraction.

Within each extraction method, we finally examined which biological functions were captured by the set of detected LC-MS features by taking the subset of LC-MS features annotated to compounds and examining any KEGG pathway of which the former are classified as members (Figure 4). In Reactor A, the positive mode data showed that the highest number of pathways were obtained from the use of MW1 and MW2 solvents (*n* = 126), followed closely by MW2 and MCW_M (*n* = 124), and a substantially lower number of pathways (*n* = 43) were implicated in the case of MCW_C. In negative mode (Figure 4), the highest number of pathways detected was found by MW1 (*n* = 126), followed by MW2, MW3 and MCW_M, from which 106 pathways were detected. Only 11 pathways were implicated from the data obtained from the MCW_C solvent mixture. On average across pathways, 21% and 23% of member compounds were recovered in positive and negative mode data, respectively, with the overall patterning of pathway recovery being highly consistent among the polar extraction solvent mixtures, and markedly different between polar and non-polar solvent mixtures (Figure 4).

For Reactor B, in positive mode, across both intracellular and intracellular compartments, more pathways were implicated, with 103, 95, 94 and 54 pathways detected in MW1, MCW_M (aqueous fraction), M and MCW_M (aqueous fraction), respectively, of which, 76 were observed from all four methods (Figure 4). In negative mode, we observed 99, 96 and 96 and 21 pathways from the MCW_M, M, MW1, and MCW_C data, respectively. Across pathways, there was a reduced recovery rate of member compounds compared to that observed in the Rector A data, with 14% and 16% being observed in positive and negative mode, respectively. The overall patterning of pathway recovery showed the same features as those of Reactor B, particularly to the difference associated with the use of polar and non-polar solvent mixtures (Figure 4).

To examine the extent of differential abundance within each extraction solvent method, we applied ANOVA to identify LC-MS features that were differentially abundant between stages of the EBPR cycle in the case of Reactor A, or those that were differentially abundant between stages and/or between compartments (extracellular or intracellular) in the case of Reactor B (see Section 4). In Reactor A for both positive and negative mode data, this analysis showed that the MW2 method gave the largest number of differentially abundant LC-MS features (including for the subset with putative compound identifications) among different stages during the EBPR cycle, followed by MW1, with the other solvent groups returning smaller numbers of differentially abundant features, or none in the case of MCW_C (Table 3 and Figure S8). 

In Reactor B, the largest number of differentially abundant LC-MS features between anaerobic and aerobic stages was found from the chloroform phase of the MCW extraction method (MCW_C, Table 3): the high number of LC-MS features (Table 2) and increased number of differentially abundant features (Table 3 and Figure S8) suggest that the microbial community from Reactor B is lipid rich, which may include a complex mixture of soluble microbial products (SMP). Therefore, the MCW solvent mixture would appear better suited to this type of community biomass, given the potential for better separation of polar and non-polar metabolites. 

## 3. Discussion

In this paper, we have explored the use of different metabolite extraction solvents for undertaking untargeted (nontarget) metabolomics analyses of complex microbial communities from activated sludge enrichment reactors: specifically, two microbial communities dominated by polyphosphate-accumulating organisms (PAOs) that facilitate the appearance of the enhanced biological phosphate removal (EBPR) phenotype. Our results suggest that the choice of extraction method needs to be carefully selected based on the microbial community under study, even among communities with similar functional phenotypes.

Our experimental design is based on sampling two distinct bioreactor communities, both of which exhibit the EBPR phenotype. Despite the similar functional phenotype, these microbial communities should not be considered as formal biological replicates due to their very different physico-chemical feeding histories and species composition, as suggested by 16S amplicon sequencing results. Accordingly, we designed our analyses to be conducted within each reactor. Within each reactor, the choice of multiple sampling events within a single feed cycle was dictated by the primary questions of solvent suitability, the need to gain data from multiple physiological states (anaerobic versus aerobic) and the sparsity of untargeted metabolomics data from microbial communities (both of these kinds and more generally). We recognise that these design choices will restrict the broader generalisability of our findings; however, our data clearly demonstrate that the metabolomic correlates of differences in physiological state can be measured in complex microbial communities, and the choice of extraction solvents needs to be evaluated for specific microbial communities.

In untargeted metabolomics studies, the chosen method for quenching and extraction is determined mostly by the physico-chemical properties of a given biological sample, such as polarity, selectivity, toxicity and inertness [12,32]. Pinu et al. provided an extensive review of quenching and extraction procedures that have been used for extraction of intracellular metabolites in cultured microbes, examining a range of chemical and/or mechanical extraction methods (including the methods we have used here in the microbial community context) [12]. Interestingly, even well-established and commonly used methods, such as cold methanol extraction [13,33,34], may require modification when used across different species: for example, when a methanol: water mixture was applied for extraction of intracellular metabolites in *Escherichia coli,* it was found that the volume ratio of methanol and water solvent impacted the effectiveness of extraction, with a recommended 80:20 *v*/*v* methanol: water mix [35]. In contrast, a 50:50 *v*/*v* ratio was chosen for *Nanoarcheum equitans* and its archaeal host *Ignicoccus hospitalis* [36] and for *Pseudomonas fluorescens, Streptomyces coelicolor* and *Saccharomyces cerevisiae* [37]. Such findings present challenging implications for performing metabolite extraction in the context of microbial communities and microbiomes due to the fact that they are comprised of many poorly understood, unculturable species, and in the present study, we chose to test multiple variations of polar solvent mixtures to obtain systematic data to study these effects in a microbiome context, as discussed further below.

Microbial community biomass also includes extra polymeric substance (EPS) or biofilm matrix [38] and complex spatially organised morphology, such as flocs or granules [10], that can present additional challenges for the selection of chemical and/or mechanical extraction methods, that may not be encountered in the analysis of well-studied cultured individual species [12]. In the present study, our sample processing method likely aggregated both cellular and matrix components of the cell pellet. The extracellular compartment studied in Reactor B was most likely close to pure supernatant, consistent with previous methods reported in the literature.

As discussed above, only a handful of untargeted metabolomics datasets are currently available in wastewater treatment microbial communities, such as activated sludge or digester sludge or from simplified communities derived from them (i.e., using continuous culture enrichment bioreactors), and this gap motivated the conduct of the present study. Previous studies exploring metabolomics of wastewater communities have used methanol: chloroform: water mixtures, for example, to capture study the extracellular metabolome and lipidome of an anaerobic digester batch [39] or oleaginous microbial communities sampled from the surface of activated sludge tanks [14,40]. In other types of microbiome samples, a wide variety of extraction solvents have been employed. For example, in soils, where polar microbiomes have been extracted using methanol: water mixtures [41], methanol: water:chloroform mixtures [41], methanol for extracellular metabolomes [42] and/or water and ethyl acetate:water for non-polar metabolites [42]. In the human gut microbiome, solvent and solvent mixtures for polar compounds have included methanol:chloroform:acetonitrile (reviewed in [2]), acetonitrile: methanol: formic acid [5], methanol: water [2,5] and, pure methanol [5] for compounds of intermediate polarity and isopropanol for both polar- and non-polar metabolites [5]. Future systematic studies on understanding the influence of extraction solvents on metabolite scope and diversity in varying microbiome contexts are clearly warranted, along with increased public availability of these raw datasets.

An additional factor, both in this setting and in microbiome research in general, is a lack of relevant biochemical knowledge of many member species due to the background problem of lack of culturability [43]. The findings of the present study are consistent with these limitations, along with clear indications that extraction method is highly biomass specific. We highlight that despite a common community level phenotype being present in the case of both communities (enhanced biological phosphorus removal), the different species profile involved in each case, and their differing flanking community members, may result in sufficiently different biomass characteristics for which different metabolome extraction solvents would be plausibly required. These results also imply that multiple extraction methods may be usefully employed, albeit at with the trade-off of increased complexity at sample handling and data analysis stages.

In our analysis, we have reported results referenced to LC-MS feature level as well as to the level of putatively identified compounds, with the former providing a view that reflects differences in bulk chemical composition, while the latter more reflects differences that can impact biological level interpretation. In regard to the latter, our analysis confirms that a major difference is observed between the use of polar and non-polar solvent mixtures. Our results suggest that biomass from each of the two reactors likely differs markedly in chemical composition, with the larger numbers of LC-MS features detected using the chloroform fraction of the MCW solvent extraction in Reactor B suggesting that this biomass has a much higher proportion of lipids than Reactor A, and which may include a complex mixture of soluble microbial products [44]. In contrast, the analysis of KEGG pathways implied by the presence of putatively annotated compounds shows relatively little variation among different polar solvent mixtures, including that from the aqueous phase of the MCW mixture. However, an additional level of interpretation can be obtained by examining the degree to which mass features, putatively annotated or otherwise, demonstrate differential abundance among different physiological states (Table 3 and Figures S8 and S9). This analysis shows that the choice of extraction solvent has a marked impact on the ability to capture the metabolome under different physiological conditions, and reflecting the combined influence of differential regulation, differential extractability and differences in chromatographic column conditions that may influence the downstream statistical estimation of differential abundance. 

This study has a number of limitations and directions for future work. As discussed above, our data convey limited generalisability due to the fact that only the EBPR cycle study has been surveyed, and, thus, repeated or parallel sampling will be priority for future analyses. Another focus of future investigations should be the examination of a wider range of non-polar solvents than the single selection used in the present study. The addition of MS2 data would permit far more confidence in compound identification, as well as from the very rapid progress being made in the analysis and interpretation of nontargeted metabolomics data [45]. Such analyses will further advance our emerging understanding of the metabolic state of these increasingly important and widely studied microbial assemblages and ecosystems.

## 4. Materials and Methods

### 4.1. Operation of the Lab-Scale SBR Enrichment Bioreactors

We sampled two laboratory enrichment bioreactor communities operated using two different protocols, each designed to enrich different PAOs, from here on described as Reactor A and Reactor B. Enrichment reactors are commonly used in environmental engineering, to both increase the relative abundance of unculturable member species and to provide a stable community for investigating a specific bioprocess or functional phenotype [46].

Reactor A was a sequencing batch reactor (SBR) with a 5.4 L working volume and was inoculated with activated sludge obtained from an existing EBPR enrichment reactor, with a protocol designed to enrich for Proteobacterial PAO species, such as those from *Candidatus* Accumulibacter. The SBR was operated with 6 h cycles, including a feeding (60 min) stage, an anaerobic stage (20 min), an aerobic stage (180 min) and a settling/decant stage (100 min). In each cycle, 2.35 L of synthetic wastewater composed of 0.53 L of solution A (containing 1.02 g/L NH_4_Cl, 1.2 g/L MgSO_4_ 7H_2_O, 0.01 g/L peptone, 0.01 g/L yeast extract and 6.8 g/L sodium acetate) and 1.82 L of solution B (0.312 g/L K_2_HPO_4_ 3H_2_O, 0.185 g/L KH_2_PO_4_, 0.75 mg/L FeCl_3_ 6H_2_O 0.015 mg/L CuSO_4_ 5H_2_O, 0.03 mg/L MnCl_2_, 0.06 mg/L ZnSO_4_, 0.075 mg/L CoCl_2_, 0.075 mg/L H_3_BO_3_, 0.09 mg/L KI and 0.06 mg/L Na_2_MoO_4_ 2H_2_O) (modified based on [47]) was introduced into the reactor continuously (in 60 min). The reactor was operated at 31 °C with an HRT and an SRT of 12 h and 7 days, respectively. The pH was controlled at 7.20–7.60 with dissolved oxygen (DO) levels maintained at 0.8–1.2 mg/L during the aerobic phase. 

Reactor B was a 5-L SBR containing a microbial consortium capable of performing EBPR and was fed with synthetic wastewater containing glucose as the main carbon source. The cycle duration was 6 h and consisted of 30 min feeding, 125 min anaerobic, 154 min aerobic, 1 min sludge discharge, 35 min settling and 15 min supernatant discharge phases. The feed composition was adapted from [47] and split into 0.3 and 1.7 L of solutions A and B fed to the reactor, respectively. Solution A contained (per litre): 1852.8 mg of glucose anhydrous, 637.5 mg of NH_4_Cl, 6.25 mg of peptone, 6.25 mg of yeast extract, 750 mg of MgSO_4_·7H_2_O, 118.75 mg of CaCl_2_·2H_2_O and 401.6 mg of N-allylthiourea to inhibit nitrification. Feed solution B contained (per litre): 73.20 mg of K_2_HPO_4_·3H_2_O, 43.65 mg of KH_2_PO_4_, 0.55 mL of trace elements solution 1 and 0.55 mL of trace elements solution 2. The trace element solutions included (per litre and adapted from [48]) are solution 1: 1.5 g of FeCl_3_·6H_2_O, 0.03 g of CuSO_4_·5H_2_O, 0.12 g of MnCl_2_·4H_2_O, 0.12 g of ZnSO_4_·7H_2_O, 0.15 g of CoCl_2_·6H_2_O and 0.1 g of EDTA disodium salt, and solution 2: 0.15 g of H_3_BO_3_, 0.18 g of KI, 0.06 g of Na_2_MoO_4_·2H_2_O. Nitrogen gas was sparged into the reactor during the anaerobic phase, while air was supplied during the aerobic phase to maintain a dissolved oxygen concentration between 0.5 and 1 mg/L. The temperature was set at 31 ± 1° C, and the pH was controlled at 7.5 ± 0.25 by using 0.5 M NaOH and 0.5 M HCl.

### 4.2. Experimental Design, Sample Collection and Extraction Procedures

We obtained samples from Reactor A and Reactor B during one 6 h cycle study (Figure 1). For Reactor A, the sampled time points were 30 min, 70 min, 95 min and 200 min relative to the start of the feeding phase. For Reactor B, the sampled time points were 30 min and 95 min relative to the start of the cycle. At each time point, a 150 mL aliquot of activated sludge was sampled. Three technical replicates were obtained from each sampled aliquot. The samples were centrifuged at 10,000 rpm for 1 min at 4 °C. Cell pellets and supernatant were separated and snap-frozen in liquid nitrogen and stored at −80 °C before lyophilisation. For Reactor A, only the pellets were used to extract intracellular metabolites, while both pellet and supernatant were used to extract extra- and intra-cellular metabolites for Reactor B. In order to normalise the sample volume, 10 mL of supernatant from each sample was lyophilised overnight (Labconco™). After lyophilisation, the samples were subjected to different metabolite extraction methods. We used up to five different extraction solutions, namely: pure methanol (M), methanol: water (50:50 *v*/*v*) (MW1), methanol: water (60:40 *v*/*v*) (MW2), methanol: water (80:20 *v*/*v*) (MW3) and methanol: chloroform: water (40:40:20 *v*/*v*/*v*) (MCW). For reactor A, we tested all five extraction solutions to examine the entire cellular component of intracellular metabolites, while for reactor B, M, MW1 and MCW were applied to extract extra- and intra-cellular metabolites. Specific extraction procedures are described below in more detail. 

#### 4.2.1. Methanol and Methanol: Water Extraction

In each case of different methanol extraction (M, MW1, MW2 and MW3), 20 mg of lyophilised pellet powder from each sample was extracted using 1 mL of extraction solution. After sonication for 10 min, the samples were centrifuged at 13,000 rpm at 4 °C for 5 min and the supernatant was collected. Extraction was repeated by adding another 1 mL of extraction solution to the pellet. After centrifugation, the supernatant from the two extractions were combined, dried by vacuum evaporator and stored at −80 °C for further analysis. The extraction procedure was the same for the extracellular metabolites by using a rotary shaker for 15 min instead of sonication. 

#### 4.2.2. Methanol: Chloroform: Water Extraction

The protocol used for the extraction of the polar and non-polar metabolites from these matrices was adapted from that of Vrhovsek et al. [49]. Briefly, 20 mg of powder from each lyophilised pellet sample was extracted using 1 mL of a mixture of water/methanol/chloroform (20:40:40). After vortexing for 1 min, the samples were put in an orbital shaker for 15 min at room temperature. Samples were centrifuged at 13,000 rpm and 4 °C for 10 min, and the upper phases constituted of aqueous methanol extract were collected. Extraction was repeated by adding another 600 µL of water/methanol (1:2) to the pellet and chloroform fractions and shaking for 15 min. After centrifugation, the upper phases from the two extractions were combined, dried by vacuum evaporator and stored at −80 °C for further analysis. The chloroform phase was also collected in a separated tube, dried by vacuum evaporator and stored at −80 °C for the analysis of non-polar metabolites. The same extraction procedure was performed for extracellular metabolites by using a rotary shaker for 15 min instead of sonication. 

### 4.3. UPLC-MS Analysis and Data Processing

Sample Preparation

Dried samples of M, MW1, MW2, MW3 and aqueous fraction of MCW extraction were reconstituted with 400 µL of water (LC-MS grade). The quality control (QC) sample was pooled from 120 µL of each sample and later used for the stability and repeatability assessment of the analysis. The QC pool was diluted down to 80%, 60%, 40%, 20%, 10%, 1% and 0% to obtain dilution QC samples. This dilution series was further used in feature extraction steps to eliminate product ions with erratic behaviour. The sample preparation step was carried out in a 96-wells plate according to the procedures described by Lewis et al. [50], with sample order being randomised prior to the automated injection step, with the aim of reducing systematic bias. A total of 150 µL of the samples was added with 150 µL of internal standards (hippuric acid-D5 and L-phenylalanine-13C9, 15N). The mixture was shaken using Thermomixer C (Eppendorf, Framingham, MA, USA) at 4500 rpm, 4 °C for 10 min. A total of 125 µL of sample mixture was aliquoted into 2 analytical plates for positive and negative modes of LC/MS analyses and placed in a 4 °C sample manager.

Organic phase extracted from MCW was resuspended with 400 µL of isopropanol/acetonitrile/water (2:1:1). A total of 100 µL of the samples was transferred to a 96-wells plate and mixed with 400 µL of lipid internal standards (LPC(9:0), PC(11:0/11:0), FA(17:0), PG(15:0/15:0), PE(15:0/15:0), PS(17:0/17:0), PA(17:0/17:0), Cer(d18:1/17:0), DG(19:0/19:0), PC(23:0/23:0), TG(15:0/15:0/15:0) and TG(17:0/17:0/17:0)). Subsequent steps of mixing and aliquoting were achieved in line with the method mentioned earlier in aqueous fraction. The preparation of QC and dilution of QC samples were performed accordingly. 

### 4.4. Metabolite Analysis Using UPLC-MS

The UPLC-MS running conditions and parameters were slightly modified from Vorkus et al. [51]. UPLC separation was conducted using an Acquity UPLC system (Waters Corp., USA) connected with HSS T3 (1.8 µm, 2.1 × 100 mm) column. Column temperature was set at 45 °C. Mobile phase A was 0.1% formic acid in water while mobile phase B consisted of 0.1% formic acid in acetonitrile. The elution gradient was set as follows: 99% A (0–0.1 min, 0.4 mL/min), 99–45% A (0.1–10 min, 0.4 mL/min), 45–35% A (10–10.15 min, 0.4–0.41 mL/min), 35–25% A (10.15–10.30 min, 0.41–0.43 mL/min), 25–15% A (10.30–10.45 min, 0.43–0.47 mL/min), 15–5% A (10.45–10.6 min, 0.47–0.55 mL/min), 5–0% A (10.6–10.7 min, 0.55–0.6 mL/min), 0% A (10.7–11 min, 0.6–0.8 mL/min), 0% A (11–12.55 min, 0.8 mL/min), 0–99% A (12.55–12.65 min, 0.8 mL/min) and 99% A (12.65–13.65 min, 0.8–0.4 mL/min). A sample loop of 2 µL was used and injection volume was set at 15 µL to ensure the full loop was filled. Mass spectrometry was performed using Xevo-G2 XS Q-ToF (Waters Corp., USA) equipped with an electrospray ionisation (ESI) source. Mass detection was scanned from 50–1200 m/z with a scan time of 0.1 s. Source temperature was set at 120 °C along with cone gas flow at 150 L/h and desolvation gas flow at 1000 L/h. Cone voltage was 20 V, while capillary voltage was 1500 and 1000 V for positive and negative mode, respectively. Leucine enkephalin (200 ng/mL in 50% acetonitrile) was used for lock mass correction with an infusion flow rate of 15 µL/min and scan frequency of 60 s. Data were acquired in MS centroid mode using MassLynx software (Waters Corp., Milford, MA, USA).

Prior to sample queueing, 5 injections of blank and 30 of the QC pool were submitted to the analysis in order to condition the LC column and MS instrument. Subsequently, 46 injections of dilution QCs were applied. The number of injections was 10, 5, 3, 3, 5, 10, and 10 times for 100%, 80%, 60%, 40%, 20%, 10%, 1% and 0% dilutions, respectively. During sample injections, pooled QC was repeatedly submitted in every 5 sample runs to determine quality of the analysis and to be employed in the data filtration process.

### 4.5. Lipid Profiling Using UPLC-MS

UPLC separation was performed using an Acquity UPLC system (Waters Corp., Milford, MA, USA) connected with CSH C18 (1.7 µm, 2.1 × 100 mm) column. Column temperature was set at 55 °C. Mobile phase A consisted of water/acetonitrile/isopropanol (2:1:1) with 20 µM phosphoric acid, while mobile phase B was made of isopropanol/acetonitrile (9:1). In both solutions, ammonium acetate was also diluted to 5 mM and acetic acid to 0.05%. The elution gradient was set as follows: 99% A (0–0.1 min, 0.4 mL/min), 99–60% A (0.1–2 min, 0.4 mL/min), 60–5% A (2–11.5 min, 0.4 mL/min), 5–0.1% A (11.5–12 min, 0.4–0.45 mL/min), 0.1% A (12–12.5 min, 0.45 mL/min), 0.1–99% A (12.5–12.95 min, 0.45–0.4 mL/min) and 99% A (12.95–14.25 min, 0.4 mL/min). A sample loop of 2 µL was used and injection volume was set at 15 µL to ensure full loop injection. Mass spectrometry was performed using Xevo-G2 XS Q-ToF (Waters Corp., USA) equipped with an electrospray ionisation (ESI) source. Mass detection was scanned from 50–2000 m/z with a scan time of 0.1 s. Source temperature was set at 120 °C along with cone gas flow at 150 L/h and desolation gas flow at 1000 L/h. Cone voltage was 25 V, while capillary voltage was 2000 and 1500 V for positive and negative mode, respectively. Leucine enkephalin (200 ng/mL in 50% acetonitrile) was continuously infused at a flow rate of 15 µL/min for mass calibration. Data were collected in centroid mode and the injection order was arranged in the same configuration as described in the previous section. The system conditioning and the number of QC samples run were the same as described above (see Section 4.5).

### 4.6. Data Processing

Feature extraction, data filtration and quality assessment were undertaken using Progenesis QI software (Nonlinear Dynamics, USA). Raw files of run-order QC, dilution QCs and all samples were imported into the software. The M + H and M-H adducts were assigned for ion detection in positive and negative modes, respectively. Peak alignment was automatically processed using one of the QC acquisitions as reference. All chromatograms were aligned to reference QC files based on retention time shifts of the major peaks. Manual adjustments were made where necessary in case of peak misalignments. There were 100–160 reference points mapped on each chromatogram. Peak picking was performed in sensitivity mode with a minimum peak width of 0.01 min. The signals acquired from hippuric acid-D5 and L-phenylalanine-13C9, 15N were used as references in the normalisation procedure of the Progenesis QI workflow. Due to the poor ionisation of some internal standards in particular modes, only detectable standards were selected as references in lipid profiling. The ions generated from PC (11:0/11:0), PG (15:0/15:0), Cer (d18:1/17:0), DG (19:0/19:0) and PC (23:0/23:0) were used as references for positive mode while FA (17:0) and PG (15:0/15:0) were selected for negative mode. To remove background noise and contaminant signal, which may be present in the dataset, all the features detected from the raw output files were filtered based on two parameters: relative standard deviation (RSD) of the features in QC runs and Pearson correlation coefficient of the features in dilution QCs. RSD value of each feature was calculated from the deviation of its ion intensity among all runs of a QC sample. The features detected with high variation in QC samples (RSD value > 30%) were removed from the dataset. Pearson correlation coefficient of each feature was generated upon linear correlation between its intensities in dilution QCs and the concentrations of dilution QCs. The rationale behind this application was that ion intensities of the compounds are supposed to decrease down according to the degrees of dilution made in dilution QCs. In this case, linear correlation between ion intensities and concentrations with a coefficient value of nearly 1.0 was observed. In this study, the features with low Pearson correlation coefficient (less than 0.8) were removed from the analysis.

### 4.7. Statistical Analysis

Statistical analyses were performed inside the R statistical computing environment [52] with use of the ggplot2 package for visualisation [53]. Principal coordinate analysis (PCoA) with Bray–Curtis similarities using ape package was performed and visualised on column mean-centred and scaled data [54].

We augmented this data visualisation with permutational multivariate analysis of variance (PERMANOVA) [31], implemented in PRIMER-e (UK), which was performed on Bray–Curtis pairwise similarities calculated using square root transformed abundance data in a sample-wise fashion as follows: (1) for Reactor A, both experimental stage (time) and solvent method were fixed, orthogonal factors, with replicates treated as a random factor nested within experimental stage, and for (2) Reactor B, solvent method, experimental stage and compartment were treated as fixed, orthogonal factors, with replicates treated as a random factor nested within experimental stage. Significance was assessed using 9999 permutations of residuals under a reduced model for the main tests. Where significant interactions between main effects were detected, appropriate post hoc tests were conducted and significance assessed as above or using Monte Carlo simulations when the number of permutations was low [30] (see Supplementary Data Files 1–4).

Within each solvent type, we performed a single-factor analysis of variance (ANOVA) of log-transformed abundance data for individual LC-MS features from positive and negative ionisation mode data, with experimental stage as the factor, followed by false discovery rate (FDR) correction using the p.adjust function in the R package stats. LC_MS features were matched against the Kyoto Encyclopedia of Gene and Genome (KEGG) compound database (the compound file, located within the KEGG ligand database; 2018 version) [55] based on accurate mass (AM) with Δppm  =  10 using a custom R script, with KEGG compound identifiers assigned if a compound match was available and these were designated as putative identifications. Annotated LC-MS features were assigned to KEGG metabolic pathways [55] based on compound membership data available in the KEGG. kgml files for KEGG pathways, which were processed using the R/Bioconductor package KEGGgraph [56]. The number of pathways identified within each mass feature dataset were visualised as a heatmap matrix using the R package ComplexHeatmap [57].

#### 16S-SSU-rRNA Amplicon Sequencing Data

From each of the two bioreactor microbial communities, biomass sampling, genomic DNA extraction, amplicon generation and sequencing were performed as previously described [58]. Analysis of raw 16S sequencing data was undertaken using the R/Bioconductor package DADA2 [59]. Relative abundance of each ASV was calculated by dividing the read count of each ASV by the sum of all ASV read counts and expressed as a percentage.

## 5. Conclusions

We used untargeted metabolomics analysis to investigate appropriate metabolite extraction solvents for different microbial communities from activated sludge enrichment reactors, demonstrating that the choice of extraction method needs to be carefully selected based on the microbial community under study, even among communities with similar phenotypes. Our approach provides direct surveys of the metabolic state of PAO enriched EBPR communities, and such data build on the key foundations for the conduct of integrated, multi-omics studies of complex microbial communities.

## Figures and Tables

**Table 1:** PERMANOVA summary of fixed terms (experimental stages *) in the model for positive mode data in Reactor A and B.

Mode	Reactor	PERMANOVA Term	DF	SS	MS	Pseudo_F	*p*-Value
Positive	A	Solvent	4	28,744	7186	45	0.0001
		Time	3	2513	838	3.8	0.0034
		Solvent × Time	12	6085	507	3.2	0.0003
		Residuals	32	5084	159	---	---
Positive	B	Solvent	2	5399	2699	140	0.0001
		Type	1	36,844	36,844	1753	0.0002
		Time	1	85	85	4.5	0.0334
		Solvent × Type	2	3965	1983	121	0.0001
		Solvent × Time	2	42	21	1.1	0.3918
		Type × Time	1	88	88	4.2	0.0251
		Solvent × Type × Time	2	42	31	1.9	0.0948
		Residuals	7	115	16	---	---
Negative	A	Solvent	4	28,884	7221	61	0.0001
		Time	3	2180	727	7.9	0.0009
		Solvent × Time	12	5894	491	4.2	0.0001
		Residuals	32	3777	118	---	---
Negative	B	Solvent	2	6181	3091	184	0.0001
		Type	1	47,733	47,733	2325	0.0001
		Time	1	80	80	4.1	0.0333
		Solvent × Type	2	6906	3453	198	0.0001
		Sovent × Time	2	44	22	1.3	0.1800
		Type × Time	1	91	91	4.4	0.0196
		Solvent × Type × Time	2	36	18	1.0	0.4429
		Residuals	7	112	17	---	---

Notes: DF: degrees of freedom; SS: sum of squares; MS: mean square. * Experimental stages: Reactor A time—stage 1 (30 min), stage 2 (70 min), stage 3 (95 min) and stage 4 (200 min). Reactor B time—stage 1 (30 min) and stage 3 (95 min); type—intracellular, extracellular.

**Table 2:** Comparison of total detected peak numbers extracted from different solvent extraction methods (positive and negative mode) for Reactor A and Reactor B.

Solvent	Total Number of Detected Peaks				Total Number of Detected Compounds			
	Reactor A		Reactor B ^a^		Reactor A		Reactor B ^a^	
	Positive Mode	Negative Mode	Positive Mode	Negative Mode	Positive Mode	Negative Mode	Positive Mode	Negative Mode
M ^b^	6934	2804	1262	1428	3025	1429	957	928
MW1 ^c^	8183	4045	1334	1816	3188	1770	1058	1104
MW2 ^d^	7699	3852	---	---	3142	1731	---	---
MW3 ^e^	7941	3604	---	---	3166	1697	---	---
MCW_M ^f^	7825	3702	1364	1735	3143	1729	1024	1067
MCW_C ^g^	2595	529	3507	514	641	68	1013	117

^a^ For Reactor B, number of detected peaks and number of detected compounds were analysed in combined intracellular and extracellular datasets. ^b^ M: pure methanol. ^c^ MW1: methanol:water (50:50 *v*/*v*), ^d^ MW2: methanol:water (60:40 *v*/*v*). ^e^ MW3: methanol:water (80:20 *v*/*v*), ^f^ MCW_M: methanol:chloroform:water (40:40:20 *v*/*v*/*v*)_aqueous fraction.^g^ MCW_C: methanol:chloroform:water (40:40:20 *v*/*v*/*v*)_choloroform fraction.

**Table 3:** Number of significant per-mass feature ANOVA tests between experimental stages * within each solvent group (at an FDR < 0.05).

Solvent	Total Number of Significant Mass Features (m/z)				Total Number of Detected Compounds			
	Reactor A		Reactor B		Reactor A		Reactor B	
	Positive Mode	Negative Mode	Positive Mode	Negative Mode	Positive Mode	Negative Mode	Positive Mode	Negative Mode
M ^a^	126	319	740	1086	112	275	483	782
MW1 ^b^	1334	719	1003	1643	460	259	761	1042
MW2 ^c^	6124	2325	---	---	2763	1246	---	---
MW3 ^d^	35	431	---	---	2538	398	---	---
MCW_M ^e^	10	0	857	1488	2	0	625	946
MCW_C ^f^	0	0	3335	501	0	0	878	93

^a^ M: pure methanol. ^b^ MW1: methanol:water (50:50 v/v). ^c^ MW2: methanol:water (60:40 v/v). ^d^ MW3: methanol:water (80:20 v/v). ^e^ MCW_M: methanol:chloroform:water (40:40:20 *v*/*v*/*v*)_aqueous fraction. ^f^ MCW_C: methanol:chloroform:water (40:40:20 *v*/*v*/*v*)_choloroform fraction. * Experimental stages: Reactor A time—stage 1 (30 min), stage 2 (70 min), stage 3 (95 min) and stage 4 (200 min). Reactor B time—stage 1 (30 min) and stage 3 (95 min); type—intracellular, extracellular.

## Data Availability

All raw data from this study have been deposited at the MetaboLights database (https://www.ebi.ac.uk/metabolights/) under accession number MTBLS884 (released date: 16 August 2019).

## Supplementary Materials

The following are available online at https://www.mdpi.com/article/10.3390/metabo11050269/s1. Figure S1. PCA analysis of non-polar metabolites extracted by MCW, Figure S2. Distribution of mass features on mass–charge ratio and retention–time axis for positive mode data from Reactor A, Figure S3. Distribution of mass features on mass–charge ratio and retention–time axis for negative mode data from Reactor A, Figure S4. Distribution of mass features on mass–charge ratio and retention–time axis for positive mode data from Reactor B, Figure S5. Distribution of mass features on mass–charge ratio and retention–time axis for negative mode data from Reactor B, Figure S6. Multi-page pdf containing total ion chromatograms (TIC) of metabolite profiles from each extraction method in Reactor A for both positive and negative mode, Figure S7. Multi-page pdf containing total ion chromatograms (TIC) of metabolite profiles from each extraction method in Reactor B for both positive and negative mode, Figure S8. Plot of adjusted ANOVA *p*-value for differential abundant mass features across the EBPR cycle by each extraction method against mean feature abundance for Reactor A, Figure S9. Plot of adjusted ANOVA *p*-value for differential abundant mass features across the EBPR cycle by each extraction method against mean feature abundance for Reactor B, Supplementary Data File 1. 16S SSU-rRNA amplicon sequencing data from Reactor A and Reactor B, Supplementary Data File 2. Post hoc comparisons of PERMANOVA analysis of metabolite profiles from positive mode data in Reactor A, Supplementary Data File 3. Post hoc comparisons of PERMANOVA analysis of metabolite profiles from negative mode data in Reactor A, Supplementary Data File 4. Post hoc comparisons of PERMANOVA analysis of metabolite profiles from positive mode data in Reactor B, Supplementary Data File 5. Post hoc comparisons of PERMANOVA analysis of metabolite profiles from negative mode data in Reactor B.
