# Supplementary material for: Influence of Extraction Solvent on Nontargeted Metabolomics Analysis of Enrichment Reactor Cultures Performing Enhanced Biological Phosphorus Removal (EBPR)
Source: Metabolites. 2021 Apr 26;11(5):269. doi: 10.3390/metabo11050269 (PMC8145293; doi:10.3390/metabo11050269)
Supplement: Supplementary file 1 [file metabolites-11-00269-s001.zip › Supplementary_Fig7_TIC_Reactor B.pdf]

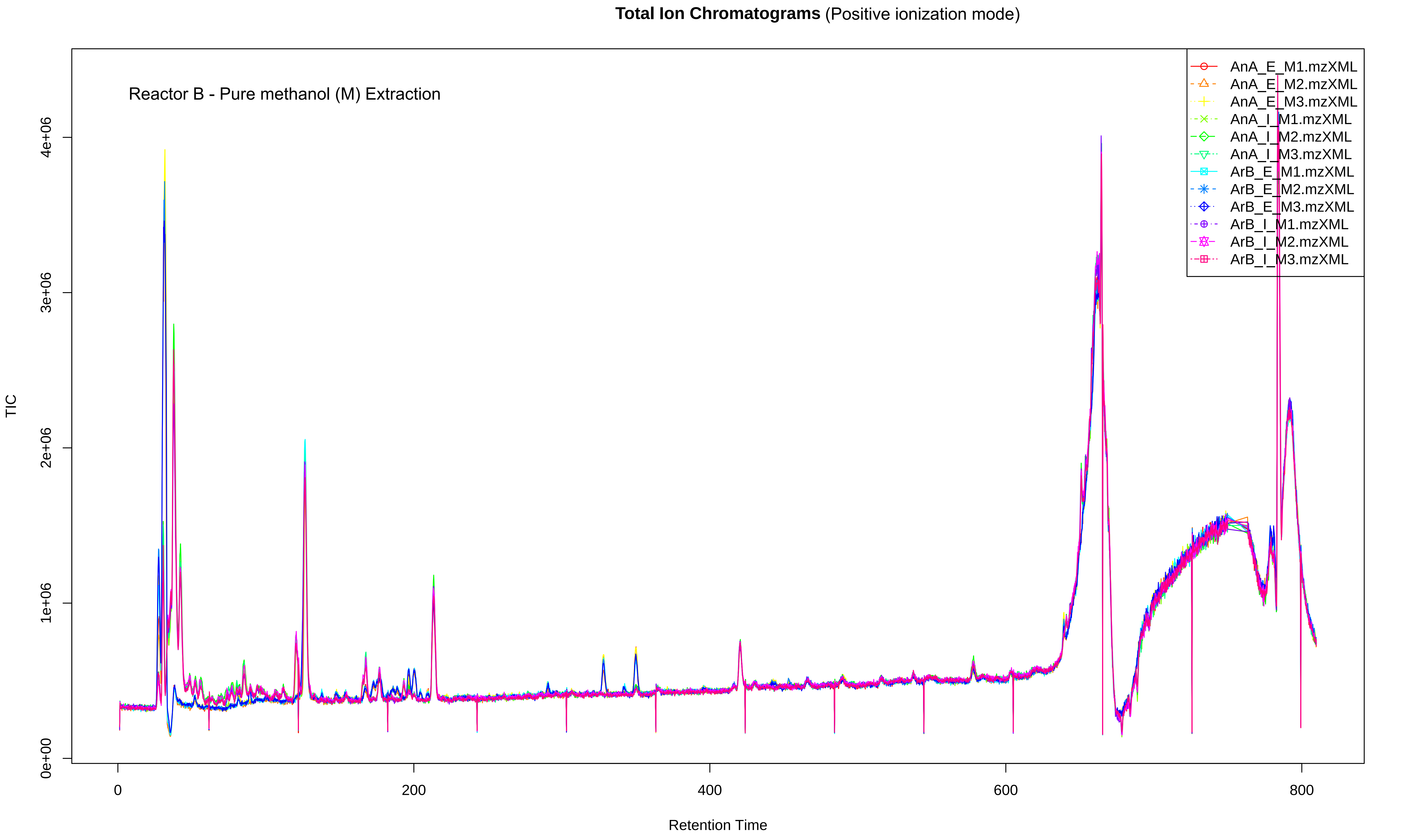
Total Ion Chromatograms (Positive ionization mode)
Reactor B - Pure methanol (M) Extraction
AnA_E_M1.mzXML
AnA_E_M2.mzXML
AnA_E_M3.mzXML
AnA_I_M1.mzXML
AnA_I_M2.mzXML
AnA_I_M3.mzXML
ArB_E_M1.mzXML
ArB_E_M2.mzXML
ArB_E_M3.mzXML
ArB_I_M1.mzXML
ArB_I_M2.mzXML
ArB_I_M3.mzXML
TIC
0e+00
1e+06
2e+06
3e+06
4e+06
0
200
400
600
800
Retention Time

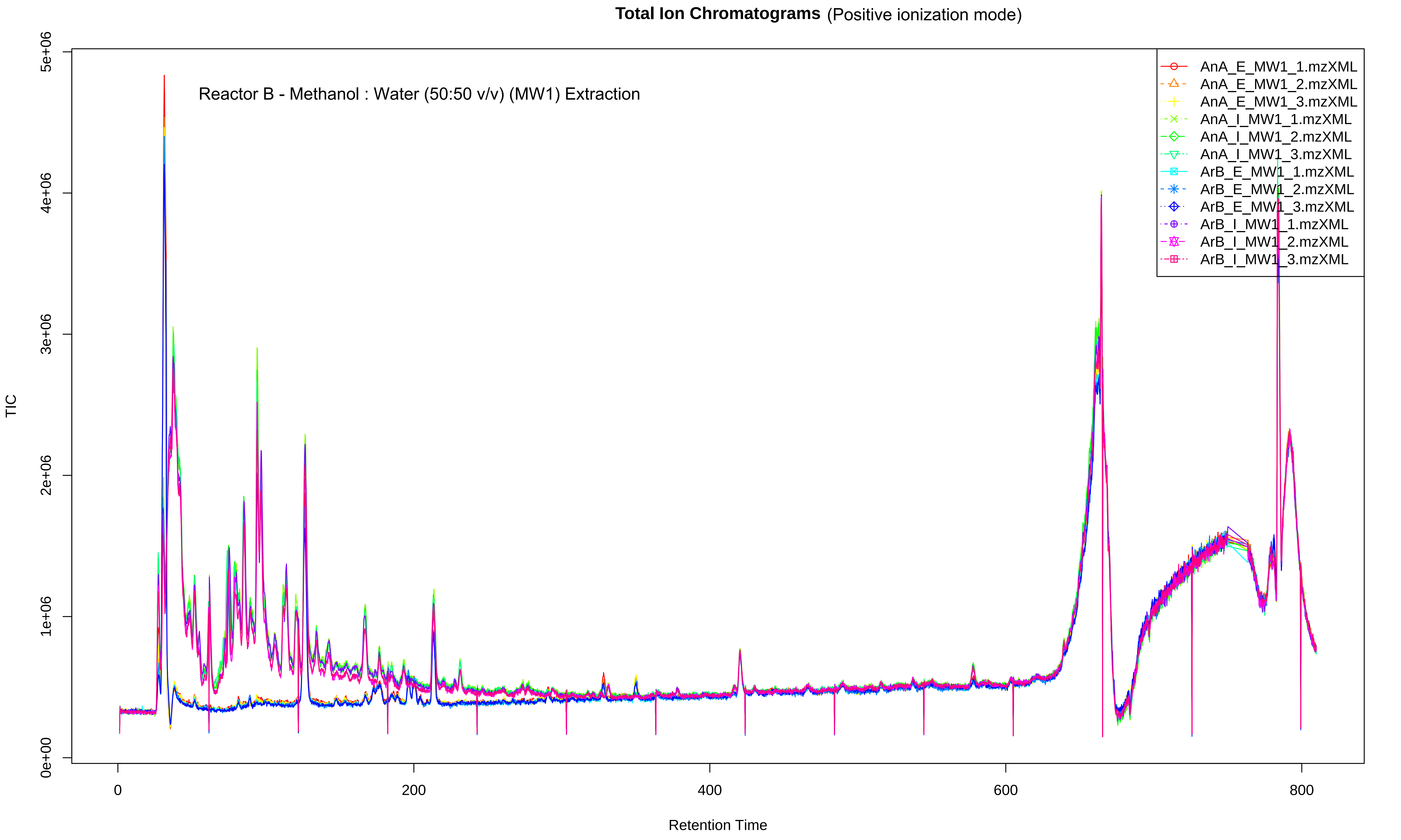
Total Ion Chromatograms (Positive ionization mode)
Reactor B - Methanol : Water (50:50 v/v) (MW1) Extraction
TIC
Retention Time
0e+00
1e+06
2e+06
3e+06
4e+06
5e+06
0
200
400
600
800
AnA_E_MW1_1.mzXML
AnA_E_MW1_2.mzXML
AnA_E_MW1_3.mzXML
AnA_I_MW1_1.mzXML
AnA_I_MW1_2.mzXML
AnA_I_MW1_3.mzXML
ArB_E_MW1_1.mzXML
ArB_E_MW1_2.mzXML
ArB_E_MW1_3.mzXML
ArB_I_MW1_1.mzXML
ArB_I_MW1_2.mzXML
ArB_I_MW1_3.mzXML

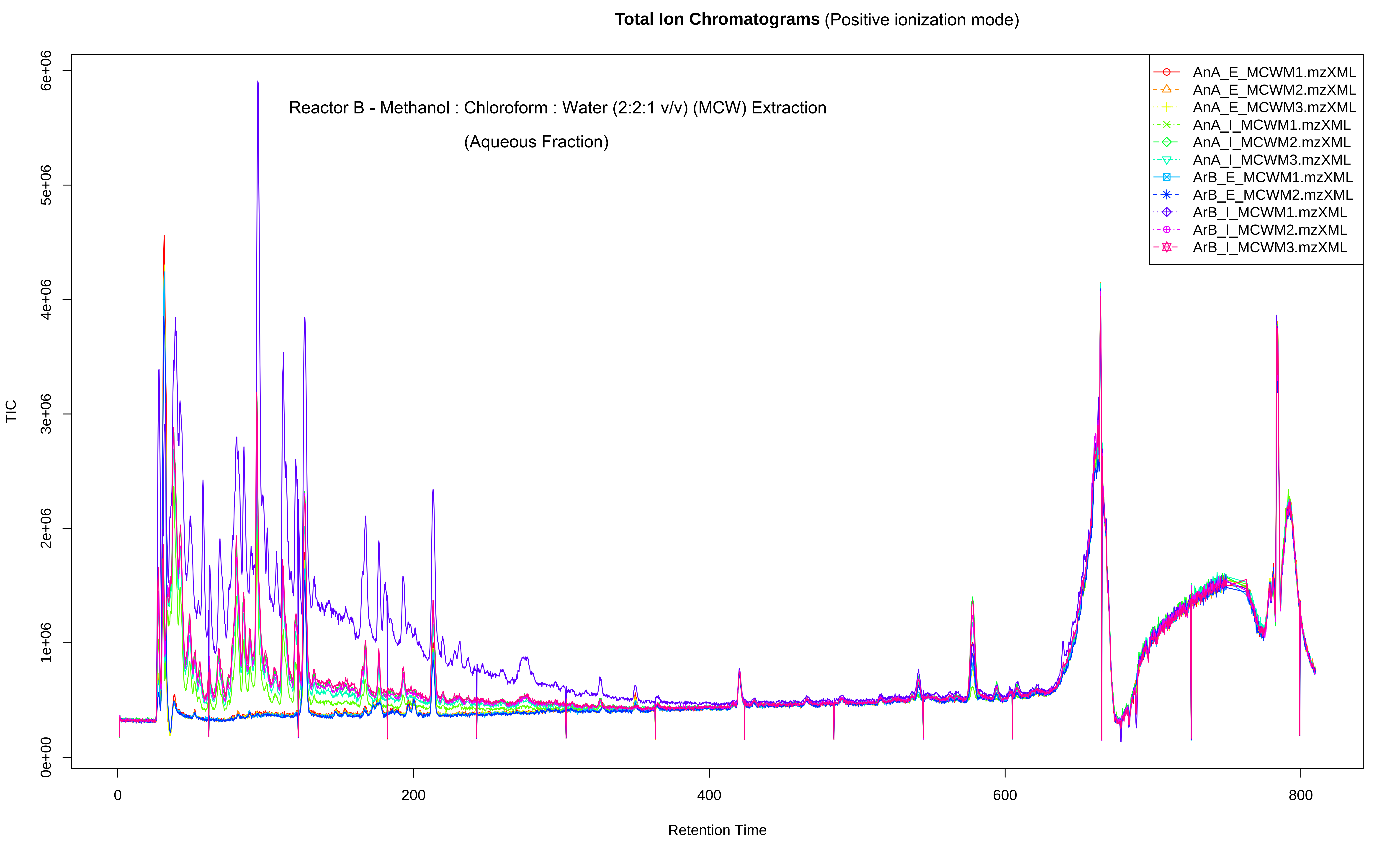

Total Ion Chromatograms (Positive ionization mode)
Reactor B - Methanol : Chloroform : Water (2:2:1 v/v) (MCW) Extraction
(Aqueous Fraction)
AnA_E_MCWM1.mzXML
AnA_E_MCWM2.mzXML
AnA_E_MCWM3.mzXML
AnA_I_MCWM1.mzXML
AnA_I_MCWM2.mzXML
AnA_I_MCWM3.mzXML
ArB_E_MCWM1.mzXML
ArB_E_MCWM2.mzXML
ArB_I_MCWM1.mzXML
ArB_I_MCWM2.mzXML
ArB_I_MCWM3.mzXML
TIC
0e+00
1e+06
2e+06
3e+06
4e+06
5e+06
6e+06
0
200
400
600
800
Retention Time

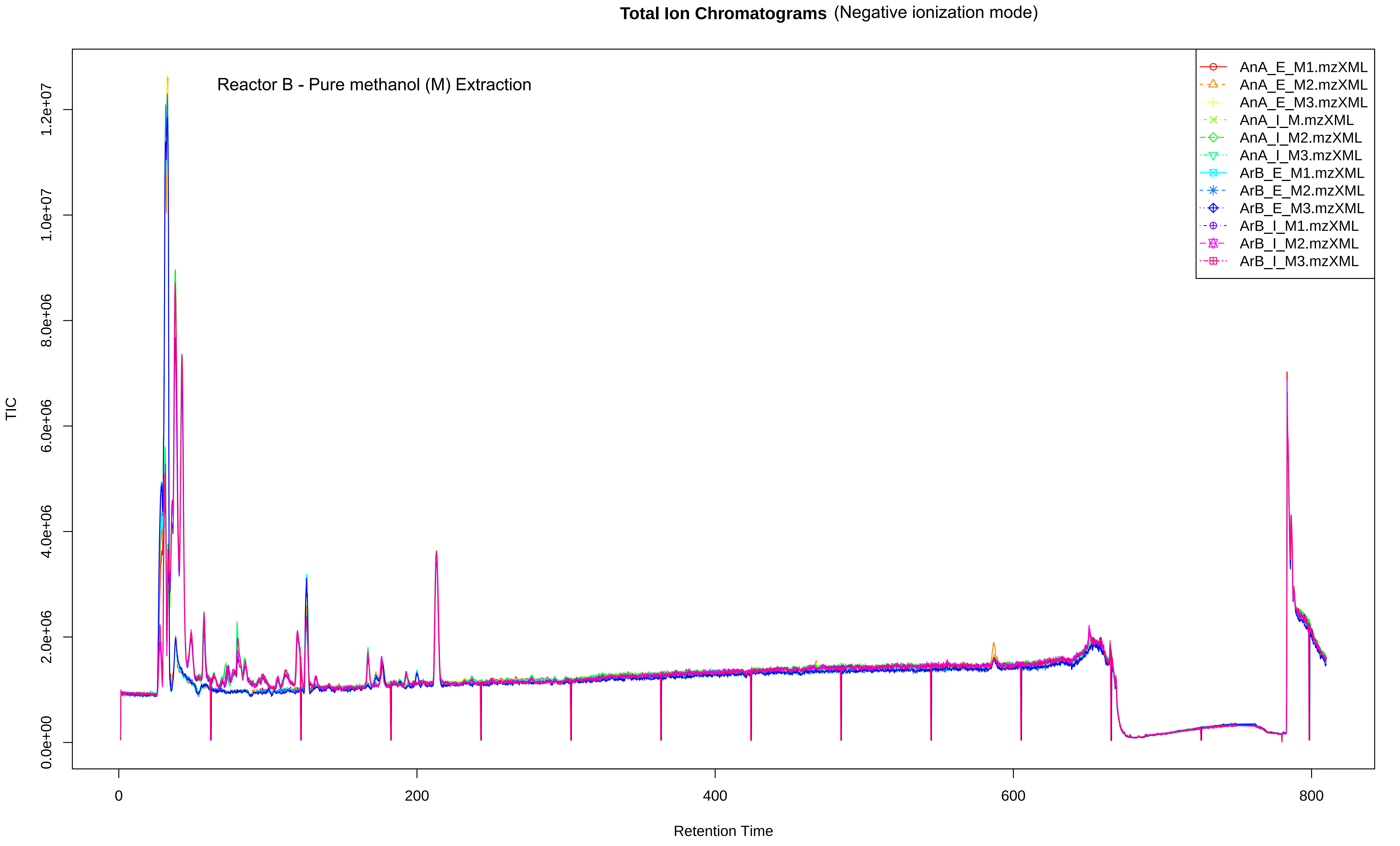
Total Ion Chromatograms (Negative ionization mode)
Reactor B - Pure methanol (M) Extraction
TIC
Retention Time
0.0e+00
2.0e+06
4.0e+06
6.0e+06
8.0e+06
1.0e+07
1.2e+07
0
200
400
600
800
AnA_E_M1.mzXML
AnA_E_M2.mzXML
AnA_E_M3.mzXML
AnA_I_M.mzXML
AnA_I_M2.mzXML
AnA_I_M3.mzXML
ArB_E_M1.mzXML
ArB_E_M2.mzXML
ArB_E_M3.mzXML
ArB_I_M1.mzXML
ArB_I_M2.mzXML
ArB_I_M3.mzXML

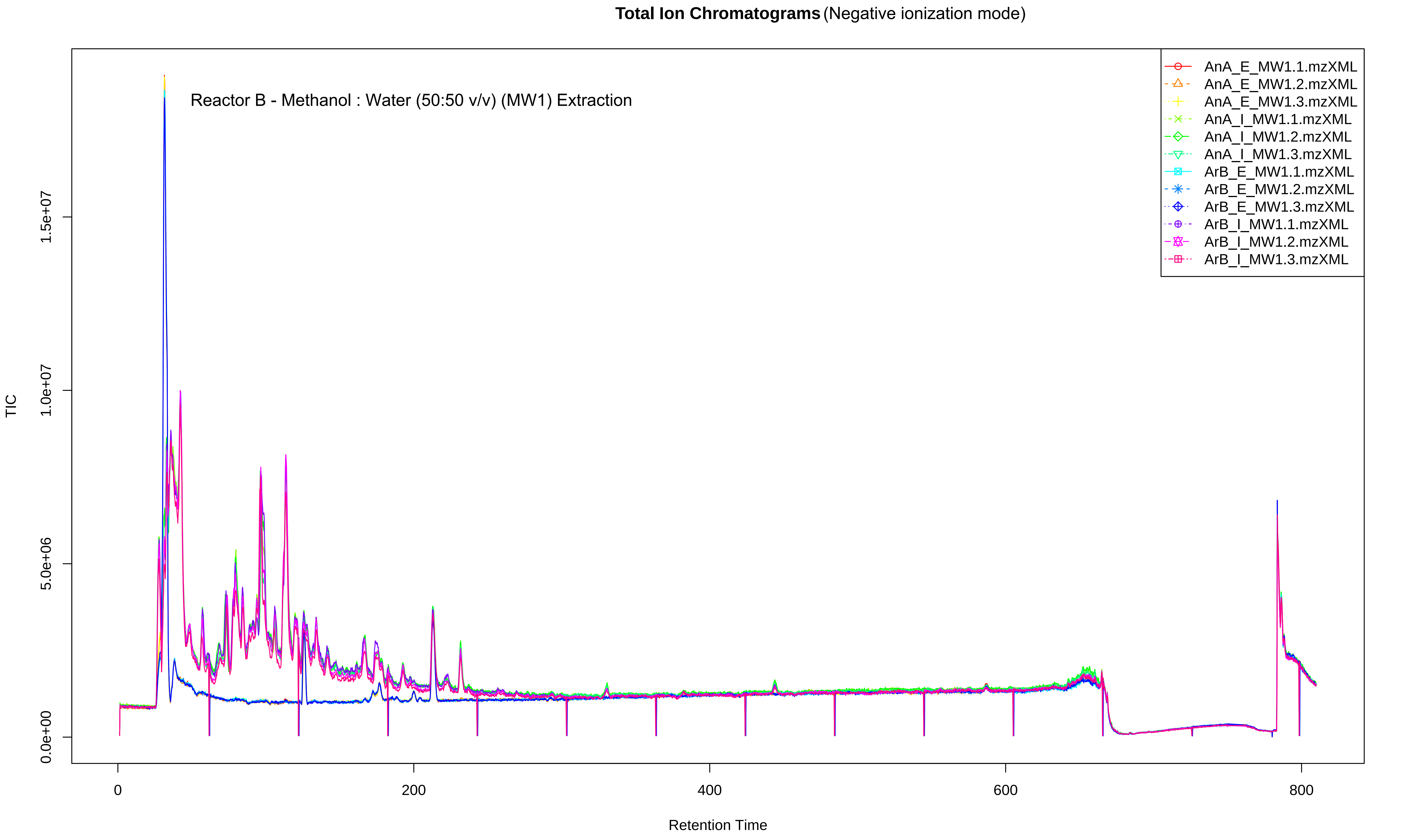
Total Ion Chromatograms (Negative ionization mode)
Reactor B - Methanol : Water (50:50 v/v) (MW1) Extraction
AnA_E_MW1.1.mzXML
AnA_E_MW1.2.mzXML
AnA_E_MW1.3.mzXML
AnA_I_MW1.1.mzXML
AnA_I_MW1.2.mzXML
AnA_I_MW1.3.mzXML
ArB_E_MW1.1.mzXML
ArB_E_MW1.2.mzXML
ArB_E_MW1.3.mzXML
ArB_I_MW1.1.mzXML
ArB_I_MW1.2.mzXML
ArB_I_MW1.3.mzXML
TIC
0.0e+00
5.0e+06
1.0e+07
1.5e+07
0
200
400
600
800
Retention Time

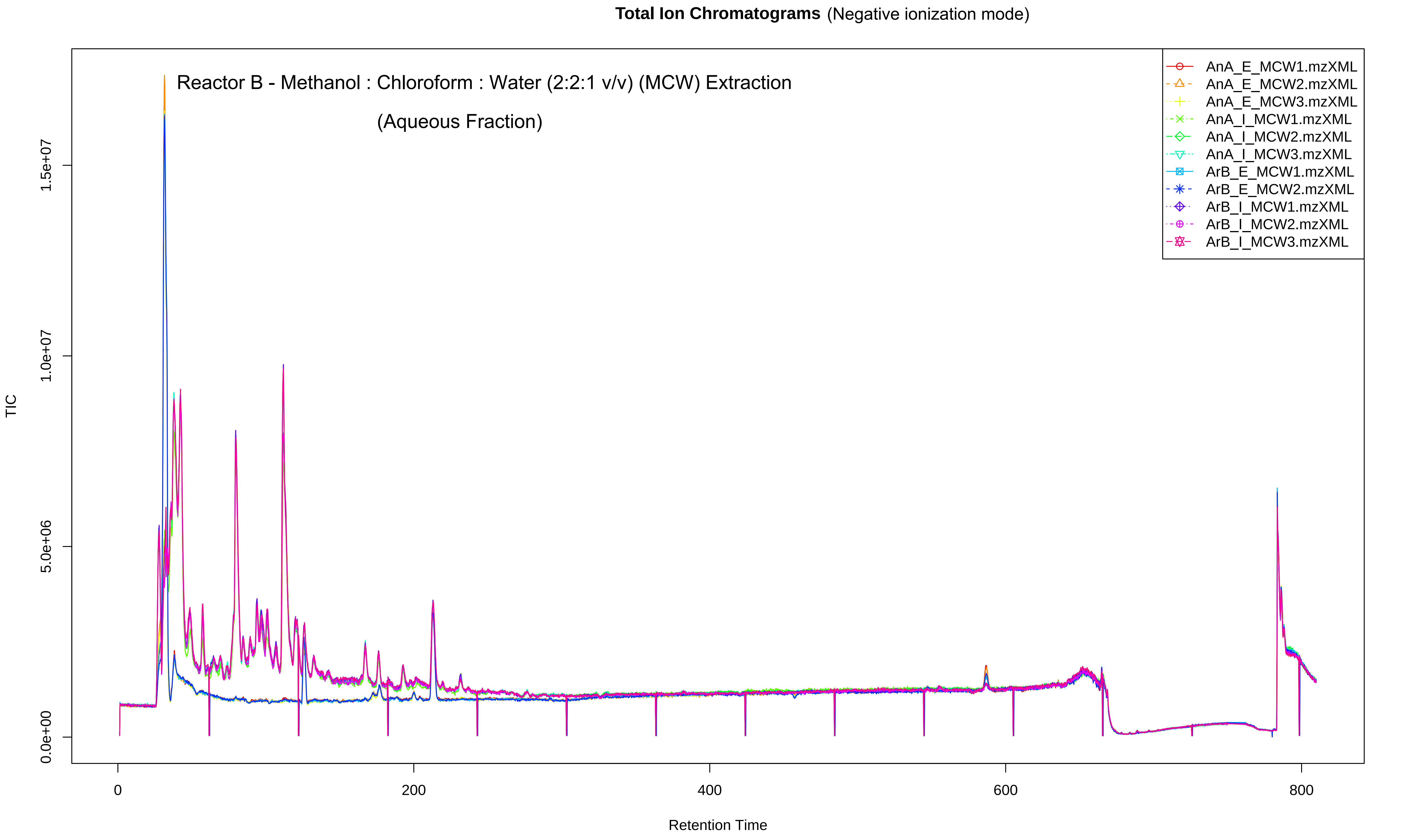

Total Ion Chromatograms (Negative ionization mode)
Reactor B - Methanol : Chloroform : Water (2:2:1 v/v) (MCW) Extraction
(Aqueous Fraction)
TIC
Retention Time
0.0e+00
5.0e+06
1.0e+07
1.5e+07
0
200
400
600
800
AnA_E_MCW1.mzXML
AnA_E_MCW2.mzXML
AnA_E_MCW3.mzXML
AnA_I_MCW1.mzXML
AnA_I_MCW2.mzXML
AnA_I_MCW3.mzXML
ArB_E_MCW1.mzXML
ArB_E_MCW2.mzXML
ArB_I_MCW1.mzXML
ArB_I_MCW2.mzXML
ArB_I_MCW3.mzXML

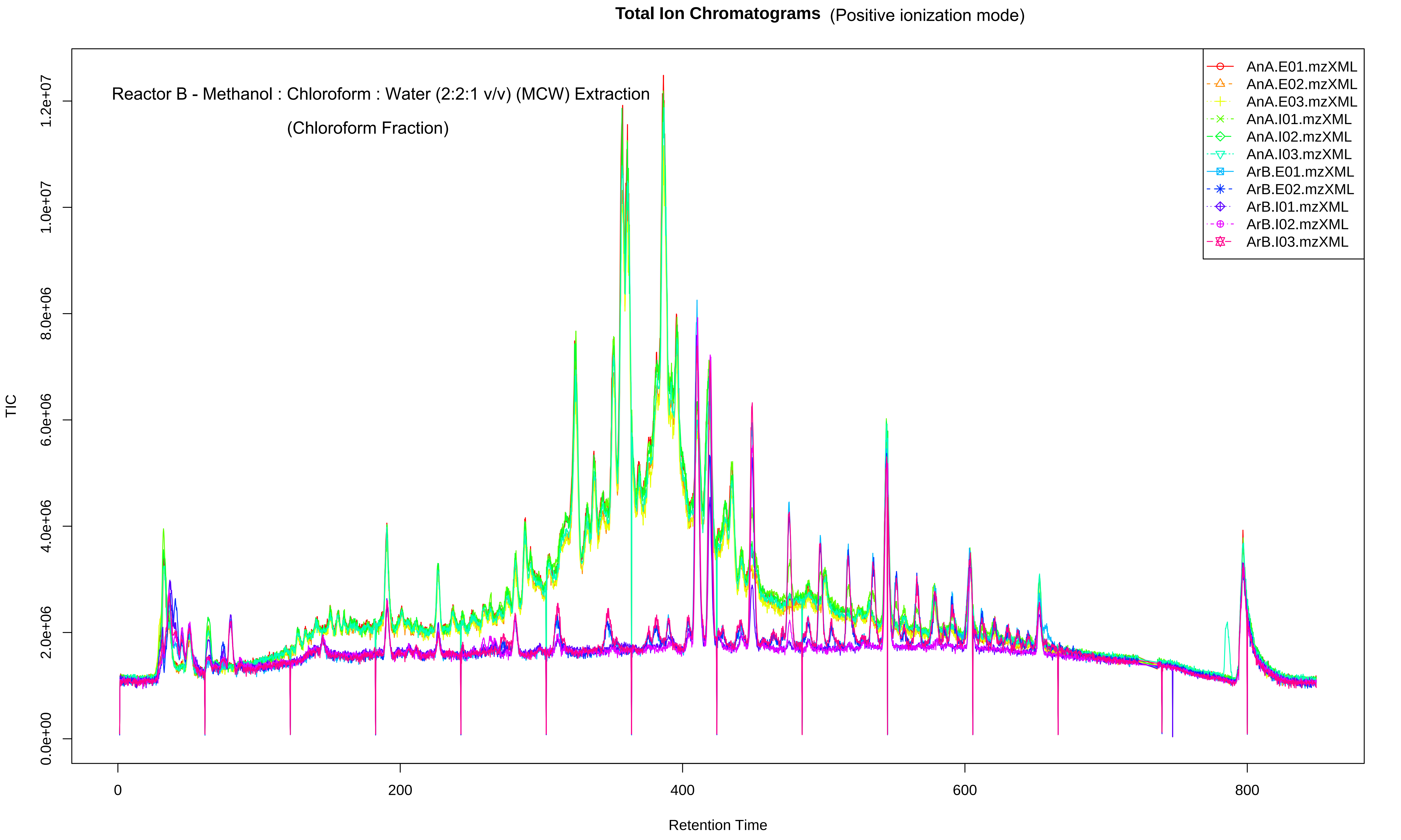

Total Ion Chromatograms (Positive ionization mode)
Reactor B - Methanol : Chloroform : Water (2:2:1 v/v) (MCW) Extraction
(Chloroform Fraction)
TIC
Retention Time
0.0e+00
2.0e+06
4.0e+06
6.0e+06
8.0e+06
1.0e+07
1.2e+07
0
200
400
600
800
AnA.E01.mzXML
AnA.E02.mzXML
AnA.E03.mzXML
AnA.I01.mzXML
AnA.I02.mzXML
AnA.I03.mzXML
ArB.E01.mzXML
ArB.E02.mzXML
ArB.I01.mzXML
ArB.I02.mzXML
ArB.I03.mzXML

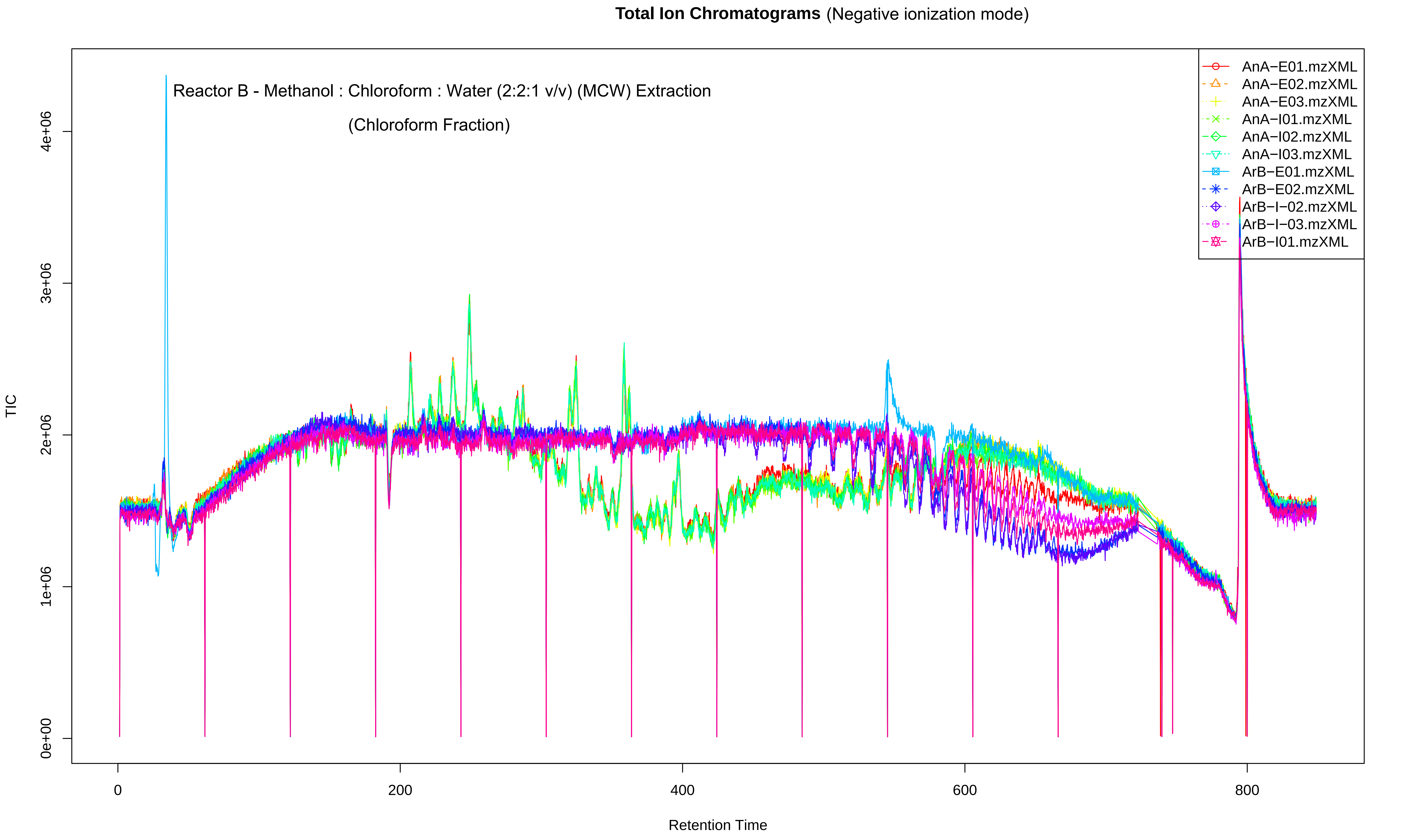
Total Ion Chromatograms (Negative ionization mode)
Reactor B - Methanol : Chloroform : Water (2:2:1 v/v) (MCW) Extraction
(Chloroform Fraction)
TIC
Retention Time
0e+00
1e+06
2e+06
3e+06
4e+06
0
200
400
600
800
AnA−E01.mzXML
AnA−E02.mzXML
AnA−E03.mzXML
AnA−I01.mzXML
AnA−I02.mzXML
AnA−I03.mzXML
ArB−E01.mzXML
ArB−E02.mzXML
ArB−I−02.mzXML
ArB−I−03.mzXML
ArB−I01.mzXML
