# Supplementary figures and images for: Influence of Extraction Solvent on Nontargeted Metabolomics Analysis of Enrichment Reactor Cultures Performing Enhanced Biological Phosphorus Removal (EBPR)
Source: Metabolites. 2021 Apr 26;11(5):269. doi: 10.3390/metabo11050269 (PMC8145293; doi:10.3390/metabo11050269)

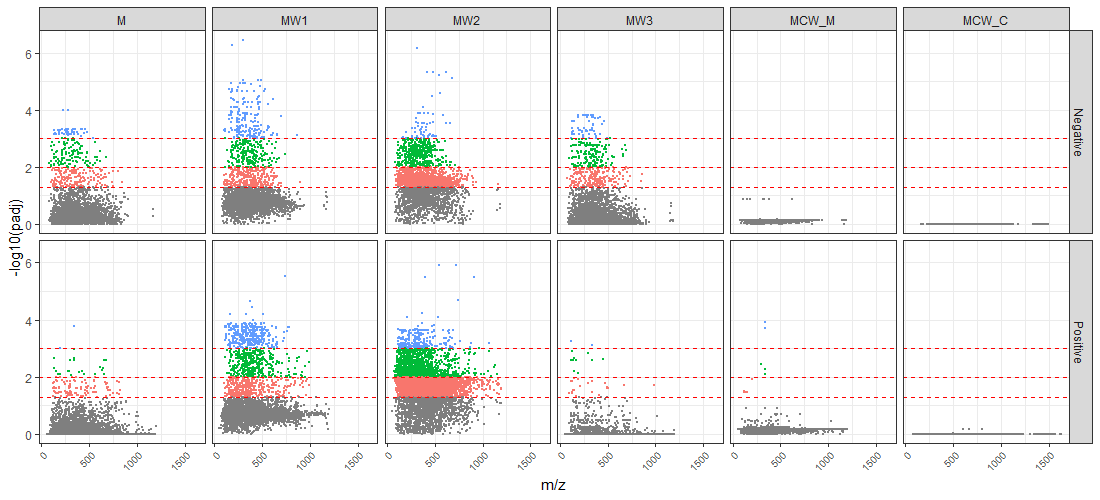

Supplement: Supplementary file 1 [file metabolites-11-00269-s001.zip › Fig S8 new.png]

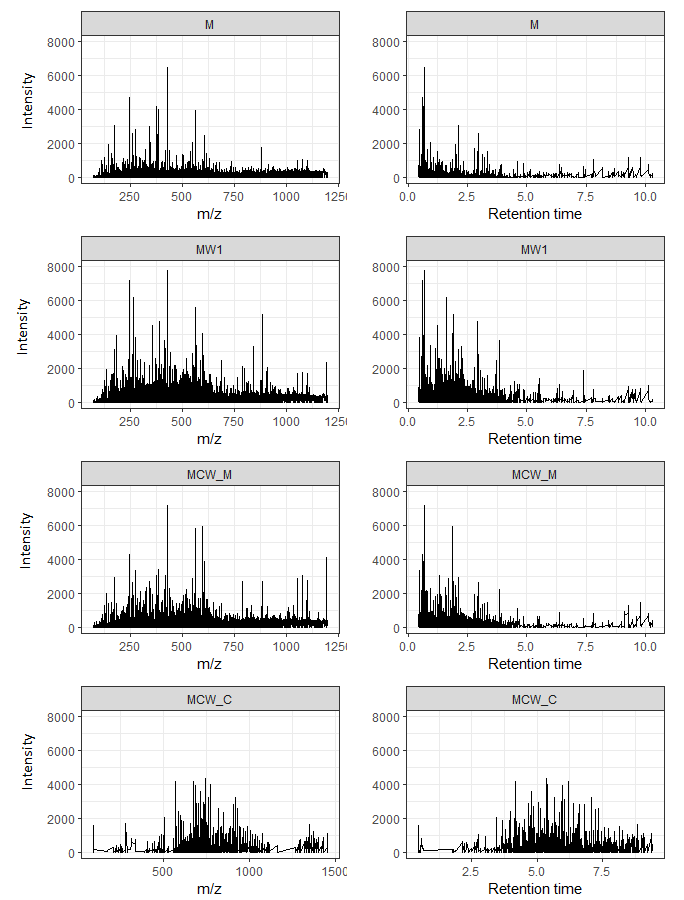

Supplement: Supplementary file 1 [file metabolites-11-00269-s001.zip › Fig S5 new.png]

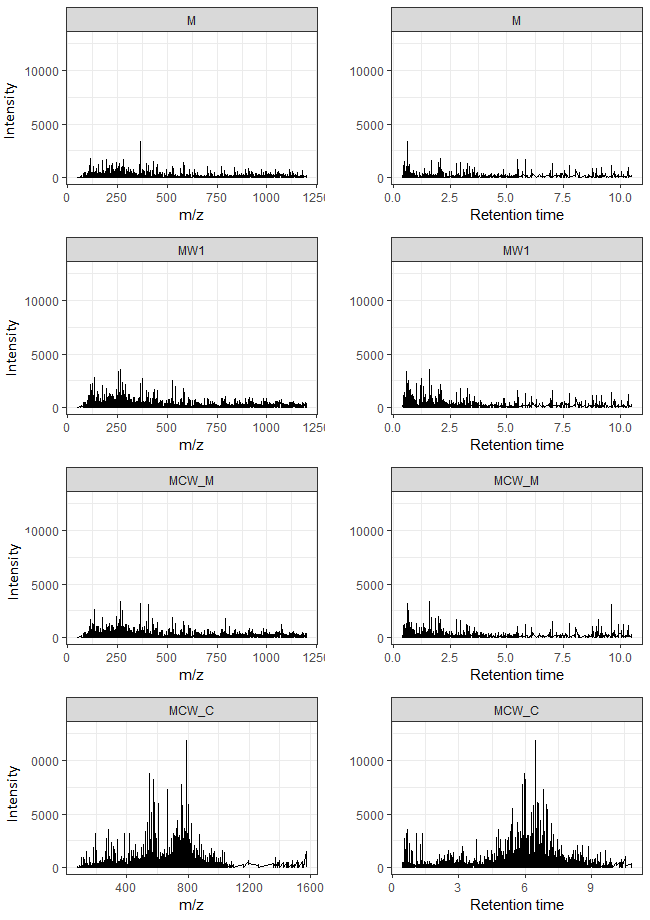

Supplement: Supplementary file 1 [file metabolites-11-00269-s001.zip › Fig S4 new.png]

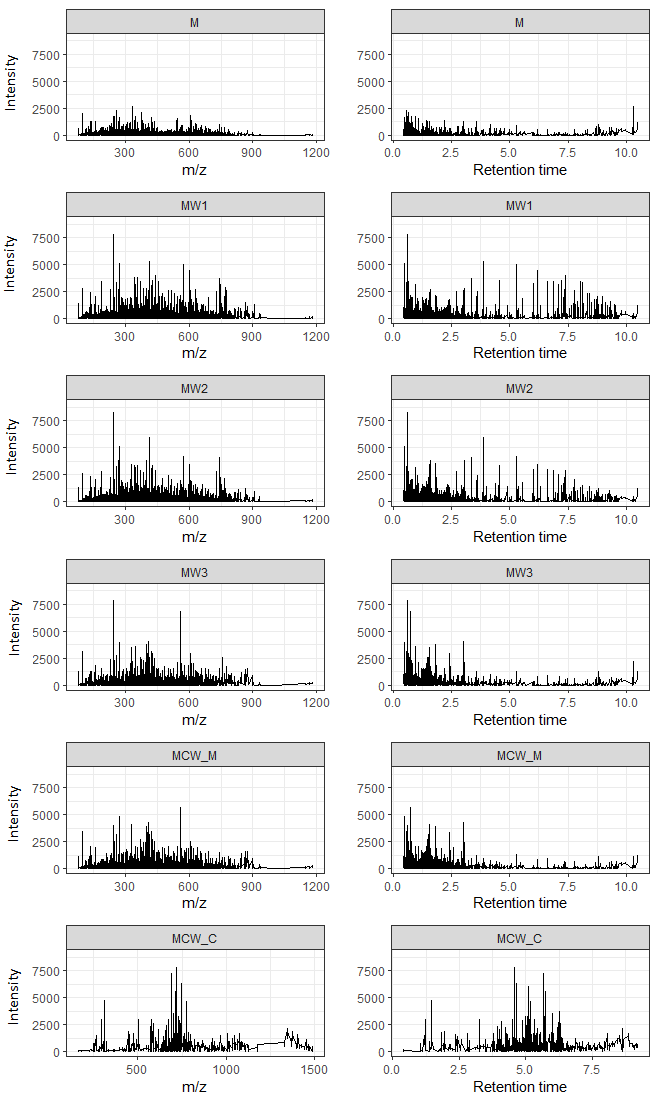

Supplement: Supplementary file 1 [file metabolites-11-00269-s001.zip › Fig S3 new.png]

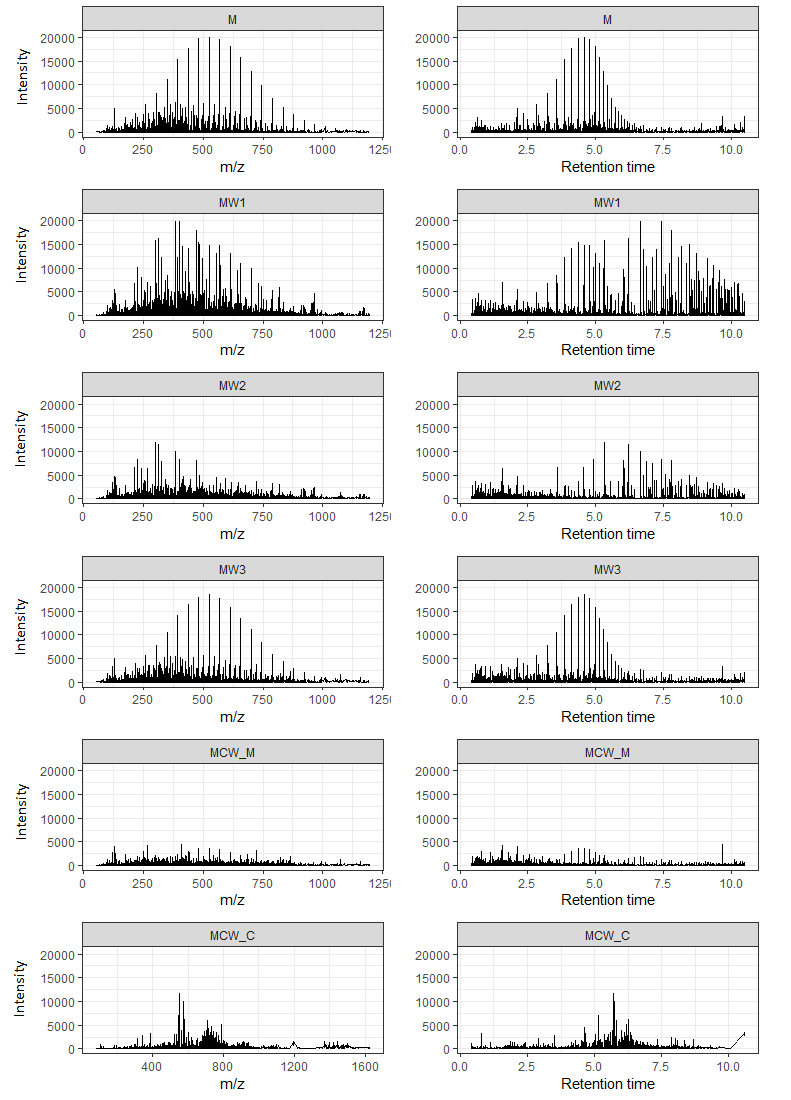

Supplement: Supplementary file 1 [file metabolites-11-00269-s001.zip › Fig S2 new.png]

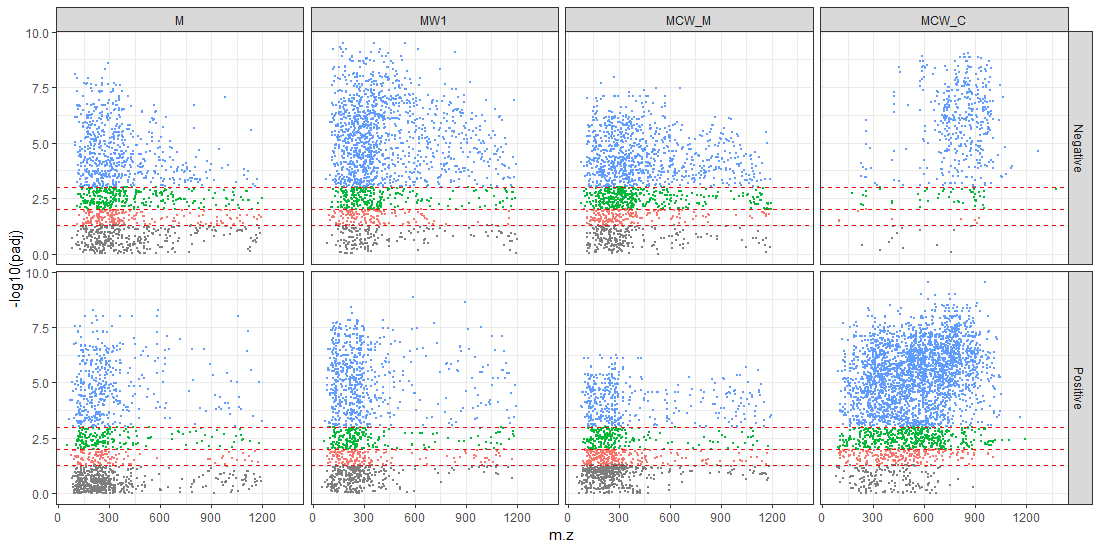

Supplement: Supplementary file 1 [file metabolites-11-00269-s001.zip › Fig S9 new.png]

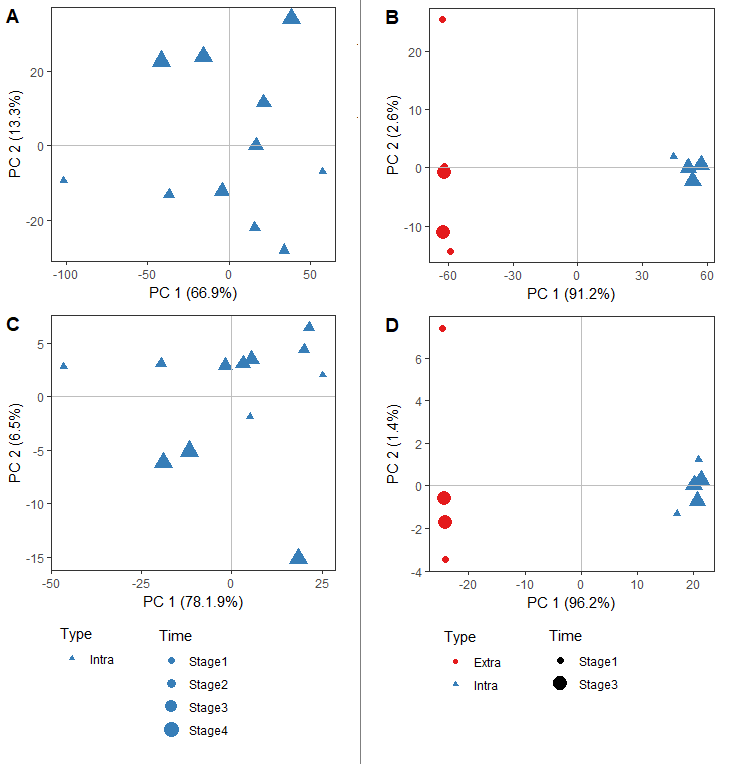

Supplement: Supplementary file 1 [file metabolites-11-00269-s001.zip › Fig S1 new.png]
